# “Oh no, the forest is burning!” cultural differences in the complex problem-solving process only under high uncertainty

**DOI:** 10.3389/fpsyg.2022.965623

**Published:** 2022-12-22

**Authors:** Willow Smith, Joanna Hermida, Christoph Dominik Güss

**Affiliations:** Department of Psychology, University of North Florida, Jacksonville, FL, United States

**Keywords:** verbal protocol, think-aloud, cross-cultural, complex problem solving, dynamic decision making, lag sequential analysis, log-linear analysis, transition frequencies

## Abstract

What do people in different cultures do when they encounter complex problems? Whereas some cross-cultural research exists about complex problem-solving predictors and performance, the process has rarely been studied. We presented participants from Brazil, Germany, the Philippines, and the United States with two computer-simulated dynamic problems, one where quick action was required – the WinFire simulation – and one where cautious action was required – the Coldstore simulation. Participants were asked to think aloud in their native language while working on these two tasks. These think-aloud protocols were digitally recorded, transcribed, and coded by coders in each country in terms of the steps involved in complex problem solving and dynamic decision making. For the current study, we developed a program to calculate transition frequencies from one problem solving step to another and analyzed only those protocols with more than 15 transitions. For WinFire, these were 256 think-aloud protocols from the four countries with a total of 12,542 statement, for Coldstore, these were 247 participants with a total of 15,237 statements. Based on previous, limited cross-cultural research, we predicted that after identifying a problem, Brazilians would make emotional and self-related statements, Germans would engage primarily in planning, Filipinos would gather additional information, and Americans would primarily state solutions. Results of latent transition analysis partially support these hypotheses, but only in the highly uncertain Coldstore situation and not in the more transparent WinFire situation. Transition frequencies were then also analyzed regarding community clusters using the spinglass algorithm in R, igraph. Results highlight the importance of process analyses in different tasks and show how cultural background guides people’s decisions under uncertainty.

## Introduction

According to Popper (1999), “All life is problem solving.” Problem solving is a part of everyday life. Complex problem solving can be defined as overcoming barriers and reaching goals in complex, dynamic, and non-transparent problem situations (e.g., Brehmer and Dörner, 1993; Funke, 2010; Dörner and Funke, 2017): Complex refers to the multitude of variables involved and their interrelatedness; dynamics refers to a state of affairs in which circumstances are changing over time; and non-transparent means that key aspects of the problem are unknown to the problem solver.

There is extensive research on complex problem solving (e.g., Frensch and Funke, 1995; Dörner, 1996; Greiff and Scherer, 2018; Schoppek et al., 2018) and dynamic decision making (e.g., Fox et al., 2013; Gonzalez et al., 2017). However, only in recent years have complex problem-solving and dynamic decision making been studied in different cultures and among different nationalities (e.g., Güss et al., 2010; Gonzalez and Martin, 2011; Lovett and Forbus, 2011; Wüstenberg et al., 2014). The present research aims to fill a gap in the existing research by testing hypotheses regarding cultural differences in complex problem-solving among Brazilian, German, Filipino, and U.S. American participants with a special focus on the problem-solving process.

Problem solving and decision making involve a series of steps: gathering information, setting goals, making plans, structuring a decision, and making a final decision (e.g., Galotti, 2002). Bransford and Stein’s (1993) classic IDEAL model proposes a similar set of steps: Identify, Define, Explore, Act, and Look back. Complex problem solving and dynamic decision making involve, however, not only cognitive, but also motivational, emotional, and self-related processes (Güss et al., 2010, 2017; Dörner and Funke, 2017), but the exact sequence of these processes and the cultural differences related to this sequence have not yet been investigated.

### Cross-cultural hypotheses regarding reactions toward problems

We expect to find cultural differences in regard to dealing with complex problem situations that are a result of knowledge that is transmitted, processed and stored by individuals (e.g., Hirschfeld and Gelman, 1994; Sperber and Hirschfeld, 1999) in a specific social, cultural, and historical context (Lave and Wenger, 1991; Cole, 1996). As Hutchins (1995, p. 354) expressed, “culture is an adaptive process that accumulates partial solutions to frequently encountered problems.” In the following sections, we attempt to derive culturally preferred patterns for different cultural groups about what to do first when confronted with a problem. Since there is a lack of research many of these hypotheses are tentative. Of course, there is also variation within cultures, but we focus here only on differences between cultures.

Prioritization of individuality and emotional expressiveness are important parts of Brazilians’ culture (Véras and Véras, 2011). Véras and Véras studied problem solving within business relationships in Brazil and China. They found that in order to successfully conduct business with Brazilians, one must build and cherish an emotional relationship before negotiating any business deals, and that Brazilians normally do not rely on strict or formal rules of communication. For example, if a Brazilian feels the need to make a statement about their own opinion, or how they feel individually, they will freely do so (Véras and Véras, 2011). Other studies (e.g., Strohschneider and Güss, 1998) have also found intense emotion expressiveness among Brazilians. We hypothesize that Brazilians will make emotional and self-related statements when encountering a problem situation more often than the other cultural groups in our study.

Germans primarily focus on orderliness and are committed to their decisions (Müller et al., 2008). In other research, German companies have been characterized as well-oiled machines (Hofstede, 2001) with a long-term time orientation (Hölter, 2013). In cross-cultural comparisons on problem solving and planning, German students have shown more detailed planning (e.g., Strohschneider and Güss, 1998). Based on these findings, we hypothesize that German participants will focus more on planning when encountering a problem situation than Brazilian, Filipino or United States participants.

Filipinos have been observed to be more hesitant to accept the reliability of information and its sources. In a study comparing decision making in the Philippines, the United States, and Hong Kong (Builtjens and Noorderhaven, 1996), Filipinos collected more information than Americans or Hong Kong residents, and delayed decision making when solving dynamic and complex problems. Thus, we expect that Filipinos will exhibit a more cautious approach to decision making (also partly due to high collectivism, Hofstede, 2001) than the other cultural groups in our study, with a focus on gathering additional information when confronted with a problem situation.

In evaluating statements, Americans focus more on what is said than on how a statement is made, which implies that Americans make explicit and direct statements when communicating and making decisions (Lisø, 2011). Other cross-cultural studies have shown that North Americans are more decisive in decision making than East Asians, i.e., they can commit to an action with more ease than East Asians (e.g., Ng and Hynie, 2014), potentially due to high United States individualism (Hofstede, 2001). These findings are in line with the contention of American philosophers John Dewey and Charles Sanders Pierce, in which pragmatism is a unique American cultural feature. Based on these findings we hypothesize that Americans will be likely to search right away for applicable solutions when confronted with a complex problem situation.

To summarize, we predict that after perceiving a problem situation, Brazilians will make primarily emotional and self-related statements; Germans will engage primarily in planning; Filipinos will gather additional information; and Americans will primarily state solutions.

## Materials and methods

### Selection of comparison countries

We attempted to select (a) countries from different continents, (b) countries that differ widely on macro-cultural variables (e.g., gross-national product, climate) and other cultural dimensions such as cultural values (Hofstede et al., 2005; Schwartz et al., 2010), (c) countries where participants have experience working with computers, and (d) countries whose languages we, the researchers, speak and where we have existing research contacts, because we intended to travel to these countries and help with the data collection and train the researchers and coders.

### Participants

In every country, participants were students from two universities who worked on both simulations (see Güss et al., 2010). In the current study we analyzed 256 WinFire think-aloud protocols and 247 Coldstore think-aloud protocols. These were 78 WinFire and 73 Coldstore protocols from Brazil, 69 WinFire and 68 Coldstore protocols from Germany, 53 WinFire and 56 Coldstore protocols from the Philippines, and 56 WinFire and 50 Coldstore protocols from the United States.

The overall age of participants ranged from 18 to 50 years old (*M* = 22.66 years, SD = 4.65). The mean age was 23.78 for the Brazilian sample (SD = 4.98), 23.01 for the German sample (SD = 3.93), 20.90 for the Filipino sample (SD = 3.11), and 22.35 for the U.S. American sample (SD = 5.76). The average age differed significantly among the four cultural groups, *F*(3, 241) = 4.28, *p* = 0.006. The Filipino sample was according to Tukey post-hoc tests significantly younger than the other samples.

Overall, 70% of the participants were female and 30% were male. In the Brazilian sample, there were 74.3% female and 25.7% male participants. In the German sample, there were 73.1% female and 26.9% male participants. In the Filipino sample, there were 65.4% female and 34.6% male participants. In the U.S. American sample, there were 67.9% female and 32.1% male participants. The gender distributions did not differ among countries, *χ*^2^ (df = 3, *N* = 246) = 1.57, *p* = 0.67.

For the current study, we only included those more elaborate think-aloud protocols that had more than 15 transitions, since we wanted to focus on transitions of the thought process and because WinFire lasted 11 min and Coldstore lasted 13 min. Answers to questions or comments of the experimenters were not coded and did not count as transitions. Other statements were not included in the number of transitions. We also had a sample from India, but transitions for most of the Indian sample were so low that we did not include it in the current study. Thus, for this study, we analyzed 256 think-aloud protocols for WinFire with a total of 12,542 statements (78 from Brazil with a total of 4,046 statements, 69 from Germany with 4,101 statements, 53 from the Philippines with 1,755 statements, and 56 from the United States with 2,640 statements). For Coldstore, there were 247 participants with a total of 15,237 statements from the analyzed think-aloud protocols (73 from Brazil with 4,742 statements, 68 from Germany with 5,696 statements, 56 from the Philippines with 1,877 statements, and 50 from the United States with 2,922 statements).

Participants were either paid for their participation in the study or received course credit. Students in all countries were from schools of social sciences, arts and sciences, and business; and 70% of the participants were psychology majors. None of the participants had taken part in other complex problem-solving (CPS) experiments prior to this study. Responding to the demographic question about work, none of the participants indicated that they had ever been employed as a firefighter – one of the simulations, WinFire, was about fighting fires.

### Instruments: Microworlds and thinking aloud

Participants worked on the two microworlds, WinFire and Coldstore. We intentionally chose these two microworlds with different task demands to not “favor” one culture over another. Winfire is a simulation that requires quick action. Coldstore is a microworld that requires cautious actions. Another factor that influenced our choice of these two simulations was the duration. Other simulations take 90–120 min and we wanted to use short simulations, as we planned recording and analyzing think-aloud protocols (Güss, 2018).

A microworld is a simulated problem in which a participant is instructed to access information and make decisions. These decisions are then implemented, the problem changes according to these decisions and other system changes, and the participant continues making decisions and solving the problem. In WinFire (Gerdes et al., 1993), participants take the role of commanding officers of a fire brigade and were instructed to save three cities and the forest from approaching fires. On the screen, participants are presented with a forest, three cities, fire-fighting trucks, helicopters, water dikes, and a cemented area. According to the criteria of microworlds, WinFire is high in complexity and dynamics, and moderate in transparency. The system consists of many variables (complexity) that are interdependent (leading to non-transparency) and that develop in a nonlinear way (dynamics). WinFire is highly complex in that it offers four main (and a few other) command options to 12 units at any given time. A person has the choice of a minimum of 4 × 12 + 4 × 11 + … + 4 × 1 = 312 alternatives. WinFire is highly dynamic because the situation changes even without any intervention from the participant, such that in 11 min, 15 fires break out at programmed times. WinFire is moderate in transparency because some information between input and output are not accessible to the participant and it is hard to predict when and where fires will start and how quickly they will spread. The success criterion in WinFire is the percentage of forests that remain protected at the end of the game; the more forest was protected the better. The simulation had 111 time intervals/cycles with 6 s each lasting in total 11 min.

In Coldstore (Reichert and Dörner, 1988, following McKinnon and Wearing, 1985), participants are required to make decisions under conditions with delayed effects. Participants take the role of a supermarket manager who must manually control the temperature of a Coldstore containing perishable goods because the automatic control device has broken down.

The Coldstore microworld can be described as low in complexity, moderate in dynamics, and low in transparency. These characterizations were validated by surveys assessing participants’ subjective perceptions of the task (Güss et al., 2005). Coldstore is low in complexity because it consists of few simulated variables and the participant has only one option to intervene which involves manipulating a control wheel. Coldstore is moderate in dynamics because it is a non-linear time-delayed response to the actions of the participant. It is low in transparency because the temperature does not immediately react to changes on the control wheel. Participants must plan and then make decisions about how to reach and maintain the optimal temperature.

Aside from the thermometer, there is a manual control (described as a control wheel), with a scale from 0 to 200, that can be used to regulate the cooling system. The closer the participant manages to bring the actual temperature to the target temperature (4 degrees Celsius or 39.2 degrees Fahrenheit), the better. The success criterion in Coldstore was the sum of the deviations between the actual temperature and target temperature. The less the temperature deviates from the target temperature, the better the performance. The Coldstore simulation had 100 cycles with 8 s each lasting in total 13 min.

While participants worked on the microworlds, they were instructed to think aloud, i.e., to say out loud everything that went through their mind without interpreting or analyzing it (see Ericsson and Simon, 1993; Güss, 2018). To analyze the verbal protocols, we decided to use a microanalytic coding system. The focus of a microanalytic coding system is on all behaviors recorded in a certain time period, that is, in our case, every single sub-sentence, such as “I send the helicopter to the fire.” In our system, raters from all countries coded single statements that enabled us to stay close to the data and leave little room for interpretation in our cross-national comparisons. The microanalytic coding system’s rigor worked for our purposes in terms of detailing the categories and differentiating these from one country to another (see Supplementary Appendix A online for the coding system with the 10 main categories and think-aloud examples;). Whereas the previously published results of these think-aloud protocols focused on country differences regarding the frequencies of the categories, for example how often did participants gather information in Brazil, India, Germany, the United States, and the Philippines (Güss et al., 2010), the current study was focused on the process. In our analysis of the protocols, we aimed to answer the question: During the problem-solving process, which step follows which step; and particularly which step follows the perception of a problem?

### Procedure

The University of North Florida Institutional Review Board together with the ethics committees in every country – where required – approved the research study in every country. Informed consent was obtained from all individual participants prior to beginning the study. The duration of each session was ~1.5 h, and all participants were tested individually with an experimenter present. The instructions and administration conditions were identical for each country. WinFire extended for 11 min; Coldstore for 13 min. After signing informed consent forms, participants received a three-page printed introduction to WinFire and a two-page introduction to Coldstore on the computer screen. These introductions were translated into German and Brazilian Portuguese using a translation-back-translation procedure as discussed by Brislin (1970). Participants played test games as practice for 3 min before each microworld to gain familiarity with the screens and commands and to practice thinking aloud. Experimenters, who were seated about three feet obliquely beside the participant, reminded the participant to continue to think aloud (i.e., “keep talking,” “continue to say what’s going through your mind”). Experimenters were instructed to give no more than seven reminders.

Experimenters were professors and an additional three to four research assistants from each country. The last author went to each of the countries and was always part of the data collection process. Participants spoke in their native tongues. Participants in the Philippines were offered the option of speaking in their native languages if they preferred, that is, Tagalog or Ilocano (for a critical discussion of the think-aloud method in cross-cultural research, see Güss, 2018). All protocols were analyzed in the language in which they were spoken and only translated to report results.

### Data analysis

Every statement in the think-aloud protocols was tape-recorded, transcribed into Microsoft Excel, and coded per idea unit by the professors, who were fluent in Brazilian Portuguese, German, English, Tagalog, and Ilocano, and by several student research assistants from each country. For example, the statement “I send helicopter 3 to the big forest fire and truck 2 to the city” has two idea units; the first referring to the helicopter, and the second referring to the truck. Raters who were not authors participated in about 10 h of training on the coding system. The following example statement has two different idea units: “I send truck 5 to the city, and then the helicopter to the water dike.” Statements triggered by the experimenter’s comments were not included in further analyses.

A coding system was developed following a theory-driven top-down and a data-driven bottom-up approach, resulting in the 10 main categories (Güss et al., 2010): (1) situation description (SD), (2) problem identification (PI), (3) formulation of goals (GO), (4) information gathering (INFO), (5) attributions and predictions (ATPR), (6) planning, decision making, and action (PLDM), (7) positive self-evaluations and emotions (SR+), (8) negative self-evaluations and emotions (SR−), (9) laughter (L), and (10) other (O).

The same coding system was used in each country for the analysis of both the WinFire and the Coldstore data (see Supplementary Appendix A). The coding system consisted of the main categories with 2–4 subcategories under each, resulting in 22 subcategories. One additional category--hypotheses about the system’s functioning – was added to the Coldstore coding. These 22 subcategories were then summarized in the 10 main categories: eight main steps (Situation description – SD, Problem identification – PI, Formulation of goals – GO, Gathering of information – Info, Attributions and predictions – ATPR, Planning, decision making, and action – PLDM, Positive self-evaluations and emotions – SR+, Negative self-evaluations and emotions – SR−), plus the Laughter and Other category (see Supplementary Appendix A). A sample of participants’ verbal protocols with their assigned codes is presented in the Supplementary Appendix online.

Raters in every country participated in about 10 h of training on the coding system where also specific examples were discussed. For example, the following statement has two different idea units: “I send the helicopter to the city / /and then the truck to the lake,” coded each with the category “Planning, Decision Making, and Action” (PLDM). Initial calculations of inter-rater reliability using Cohen’s Kappa (Cohen, 1960) were conducted on a sample of think-aloud protocols from each country to check the credibility of the coding system for the think-aloud protocols of WinFire and Coldstore and the coders’ training (see Güss et al., 2010). According to Fleiss (1981), Kappas over 0.75 are excellent, between 0.60 and 0.75 are good, and between 0.40 and 0.60 are fair. Inter-rater reliability was 0.59 (Brazil), 0.60 (United States), 0.66 (Philippines), and 0.83 (Germany). Disagreements were resolved through discussion among the coders that was aimed at seeking consensus. The resulting Kappas ranged from good to excellent (Fleiss) and were considered satisfactory for such a complex coding system and such heterogeneous samples.

**Figure 1:**
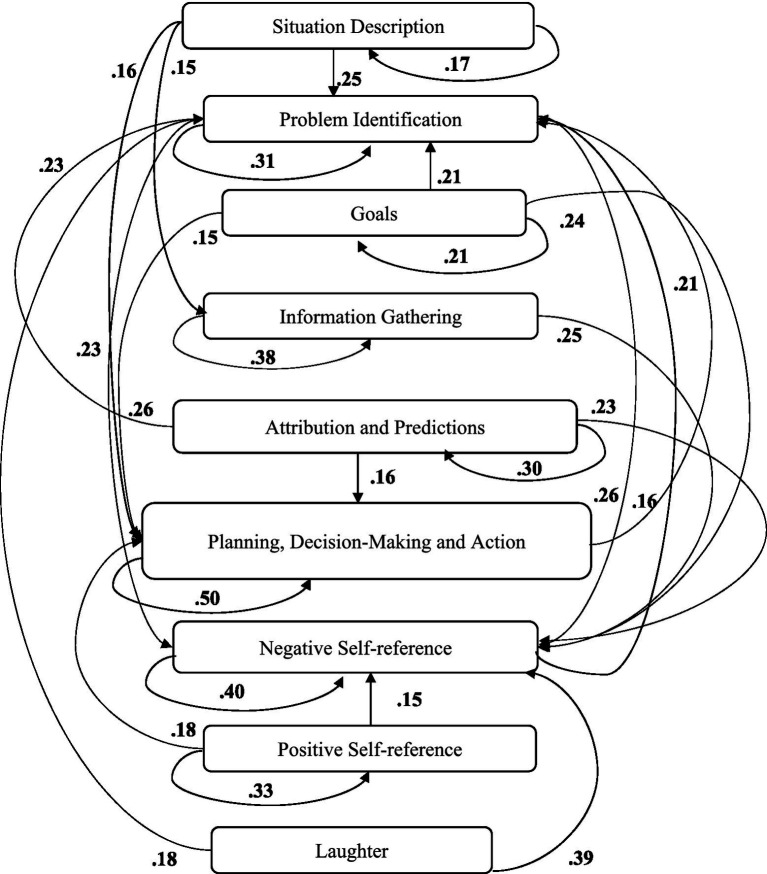
Brazil – WINFIRE conditional probabilities greater 15 percent with significant z-score values.

**Figure 2:**
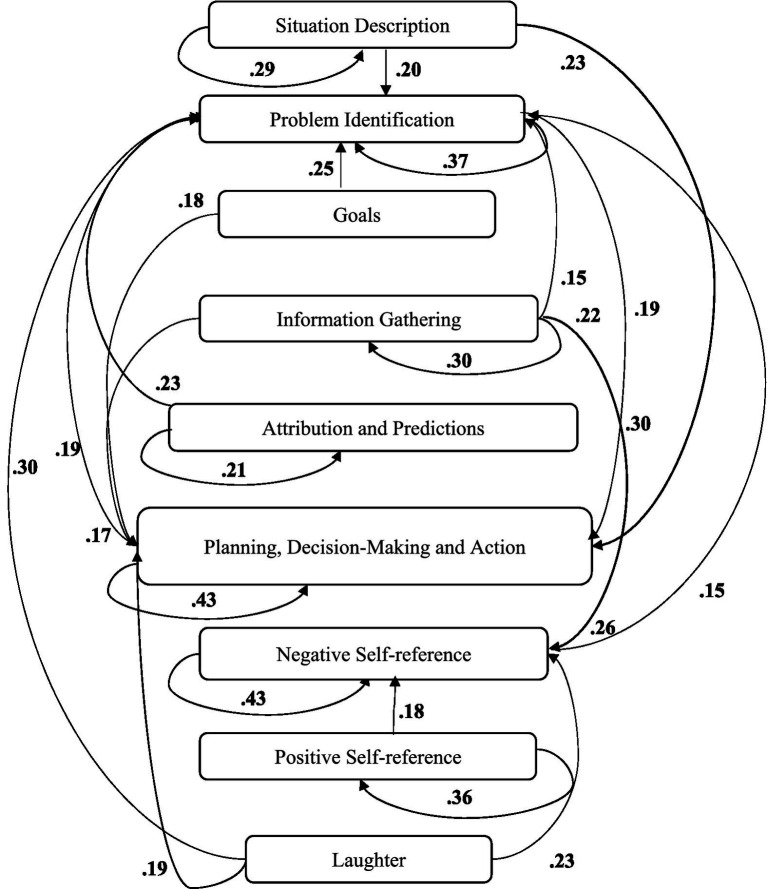
Brazil – COLDSTORE conditional probabilities greater 15 percent with significant z-score values.

**Figure 3:**
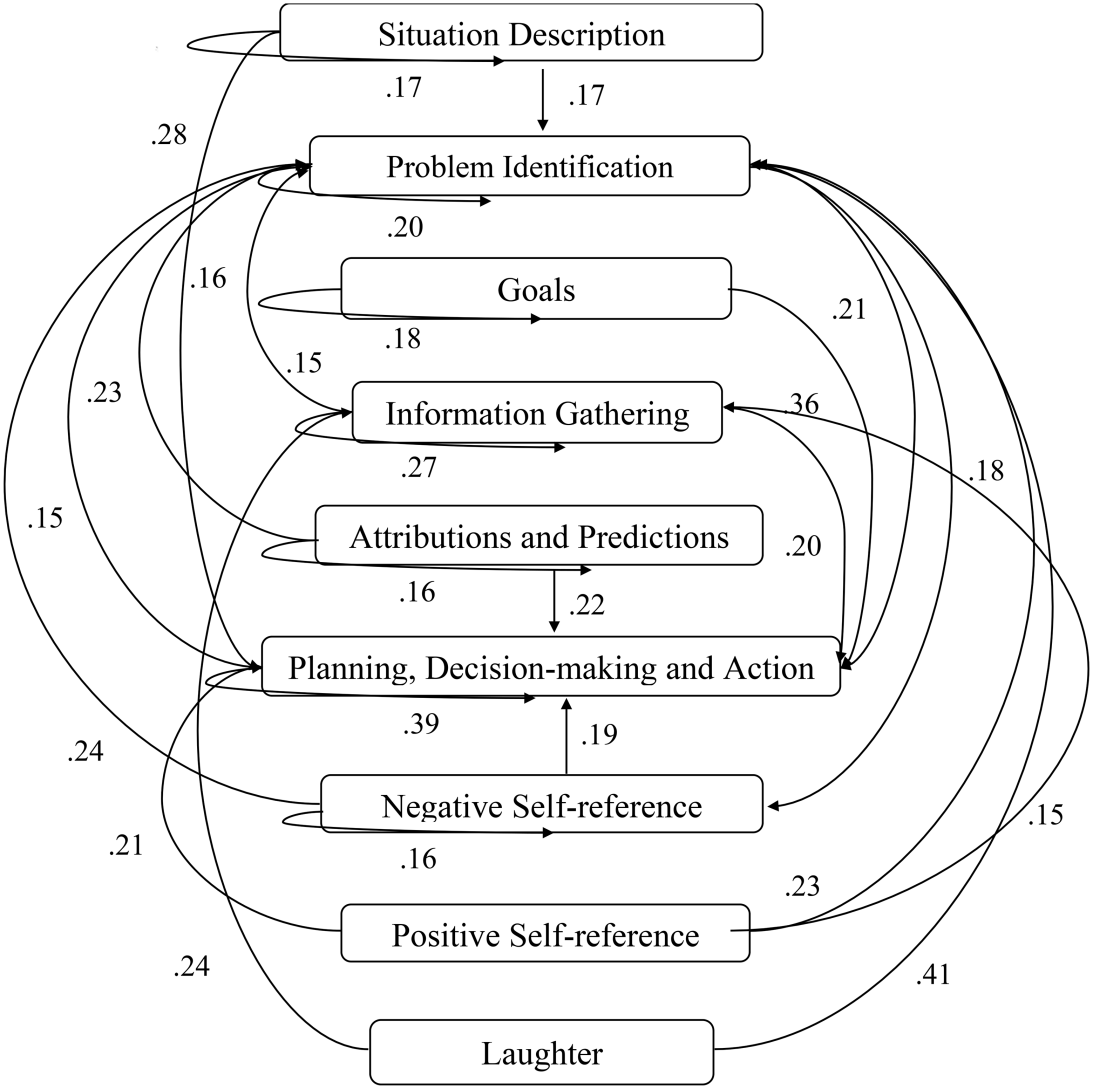
Germany – WINFIRE conditional probabilities greater 15 percent with significant z-score values.

**Figure 4:**
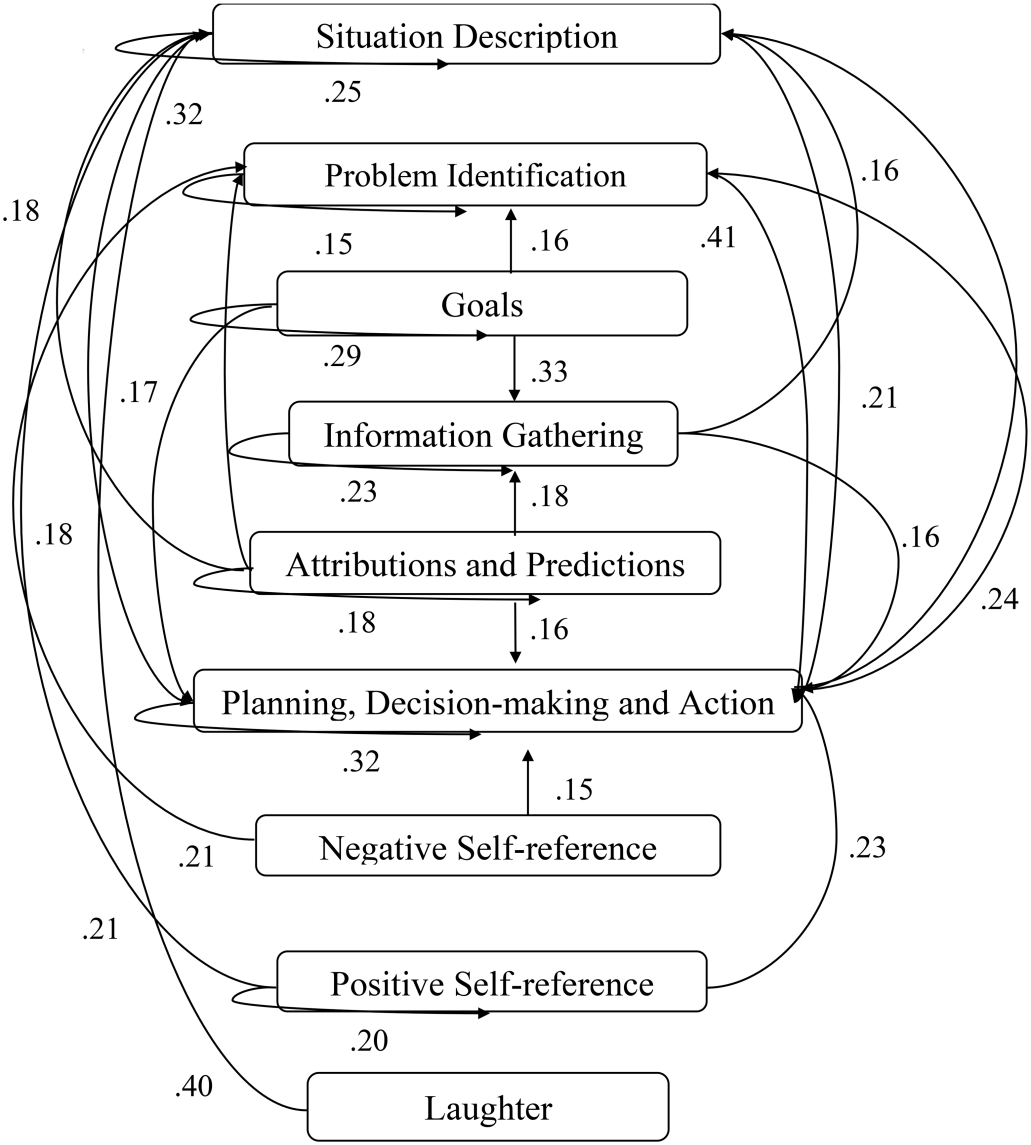
Germany – COLDSTORE conditional probabilities greater 15 percent with significant z-score values.

**Figure 5:**
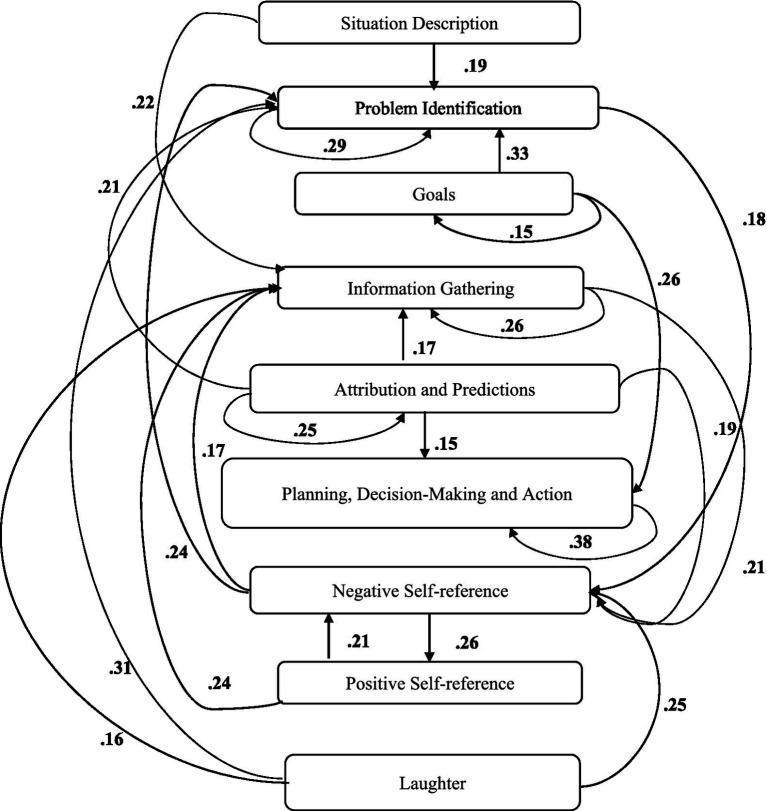
Philippines – WINFIRE conditional probabilities greater 15 percent with significant z-score values.

**Figure 6:**
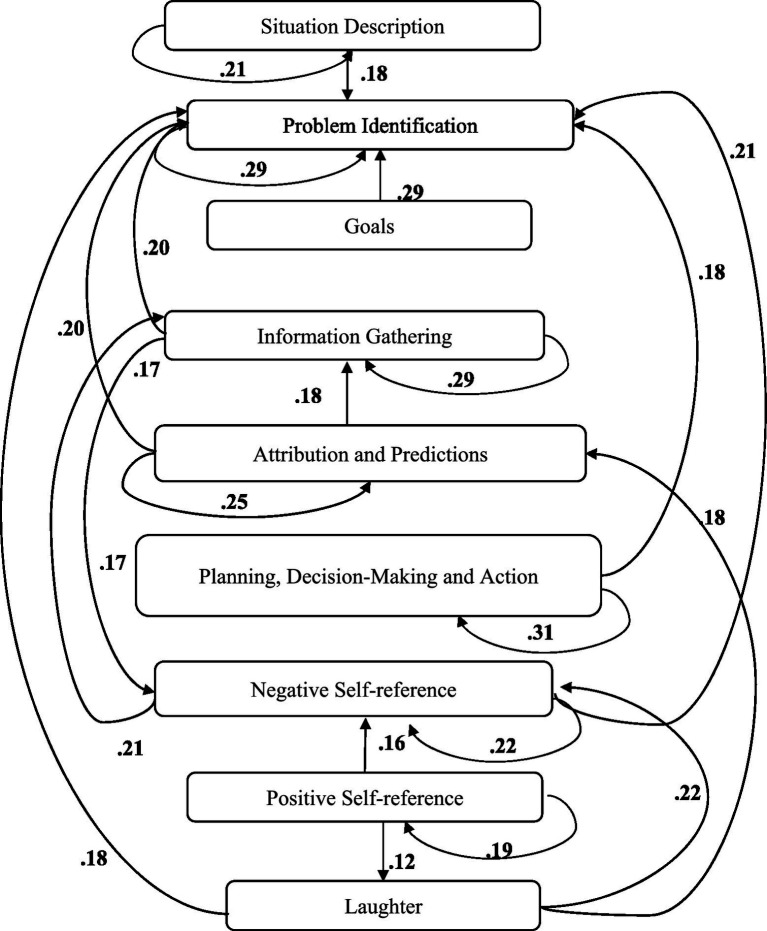
Philippines – COLDSTORE conditional probabilities greater 15 percent with significant z-score values.

**Figure 7:**
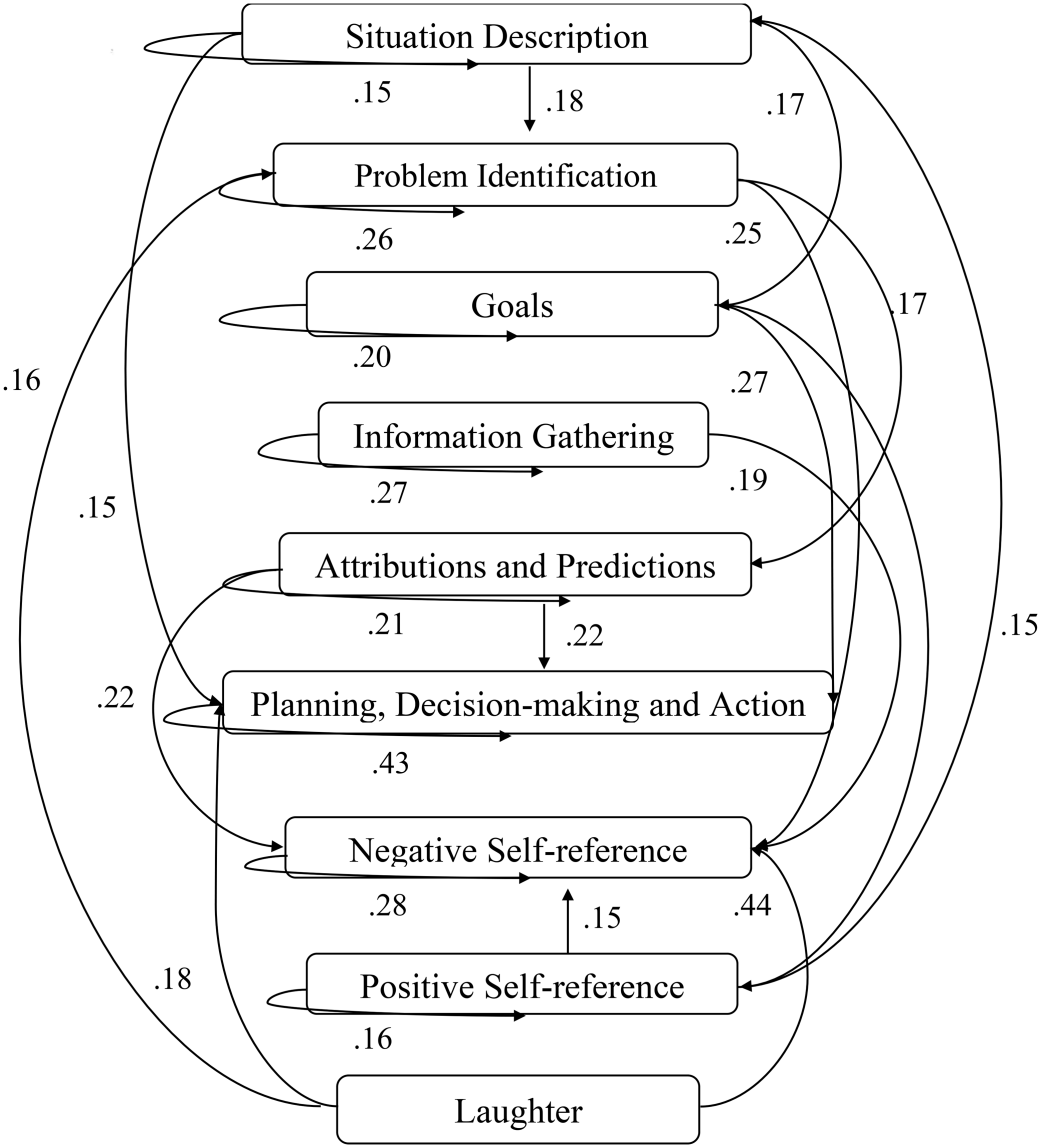
United States – WINFIRE conditional probabilities greater 15 percent with significant z-score values.

**Figure 8:**
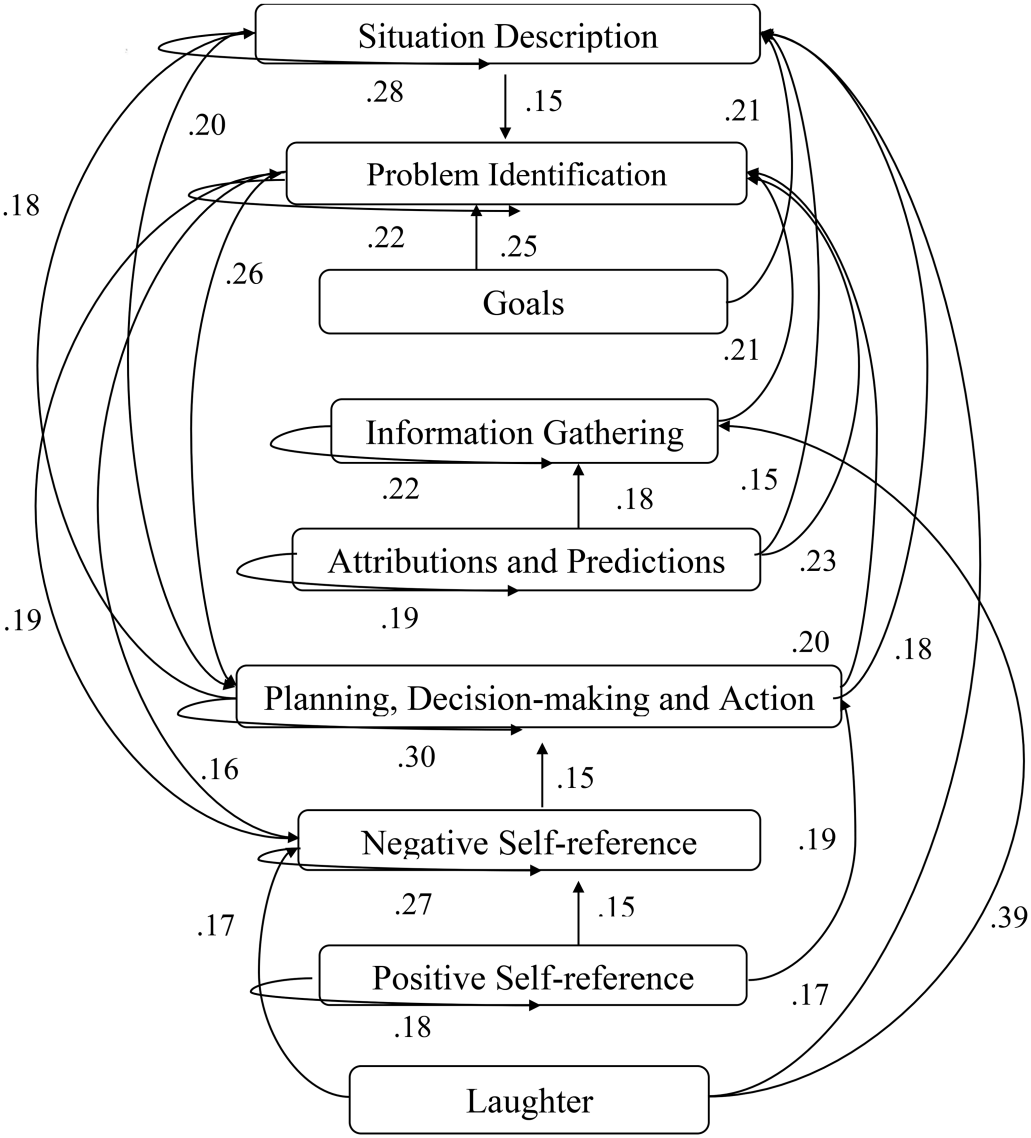
United States – COLDSTORE conditional probabilities greater 15 percent with significant z-score values.

A computer program was created by Bhattacharya (2019) that read the transcribed think-aloud and coded think-aloud files of every participant saved in the Excel program for WinFire and Coldstore and then calculated the transition frequencies for each step. For example, how often were goals (GO) formulated after a problem was identified (PI)? How often did planning (PLDM) happen after a problem was identified (PI)? All statements and transitions that related to responses of experimenter interventions or “other” statements (e.g., repeating exact same statement, the first statement as a response to experimenter statement, “Mmmmh,” “Ahhh”) were excluded. One output file shows the transitions among the 22 subcategories, the other output file shows the transitions among the nine main categories for each WinFire and Coldstore (see Coding System in Supplementary Appendix A). The transitional probability (TP) – also called conditional probability – from any category x to another category y is given by TP (x → y) = frequency (xy)/frequency (x). These analyses are also called lag analysis or latent transition analysis (Collins and Lanza, 2010). Transition frequencies were summarized and averaged for each country for WinFire and Coldstore (see Figures 1–8). The figures show only transitions >15% for easier comprehension. In order to calculate significant transitions (see Gottman and Roy, 1990), we also calculated observed frequencies, conditional probabilities, expected frequencies, and z-scores for each microworld and each country (Data for this study can be found in the Supplementary material section, Supplementary Appendix B). Z-scores less than or equal to the value of −1.645 and z-scores higher or equal to the value of 1.645 are significant at the 0.05 level.

We then used these data files on conditional/transitional probabilities to represent communities in a graph. “A community is defined as a subset of nodes within the graph such that connections between the nodes are denser than connections with the rest of the network” (Radicchi et al., 2004). We used the spinglass algorithm in R, igraph, to graph these communities (Reichardt and Bornholdt, 2006, see Supplementary Appendix C for the R code).

## Results

The four hypotheses were as follows: After presenting participants with problem scenarios, Brazilians will emphasize making emotional and self-related statements (SR−), Germans will engage primarily in planning (PLDM), Filipinos will focus on gathering additional information (INFO), and Americans will primarily state solutions (PLDM). Statement transitions over 15% for each country are shown in Figures 1–10 for WinFire and Coldstore (Figures 11–24).

**Figure 9:**
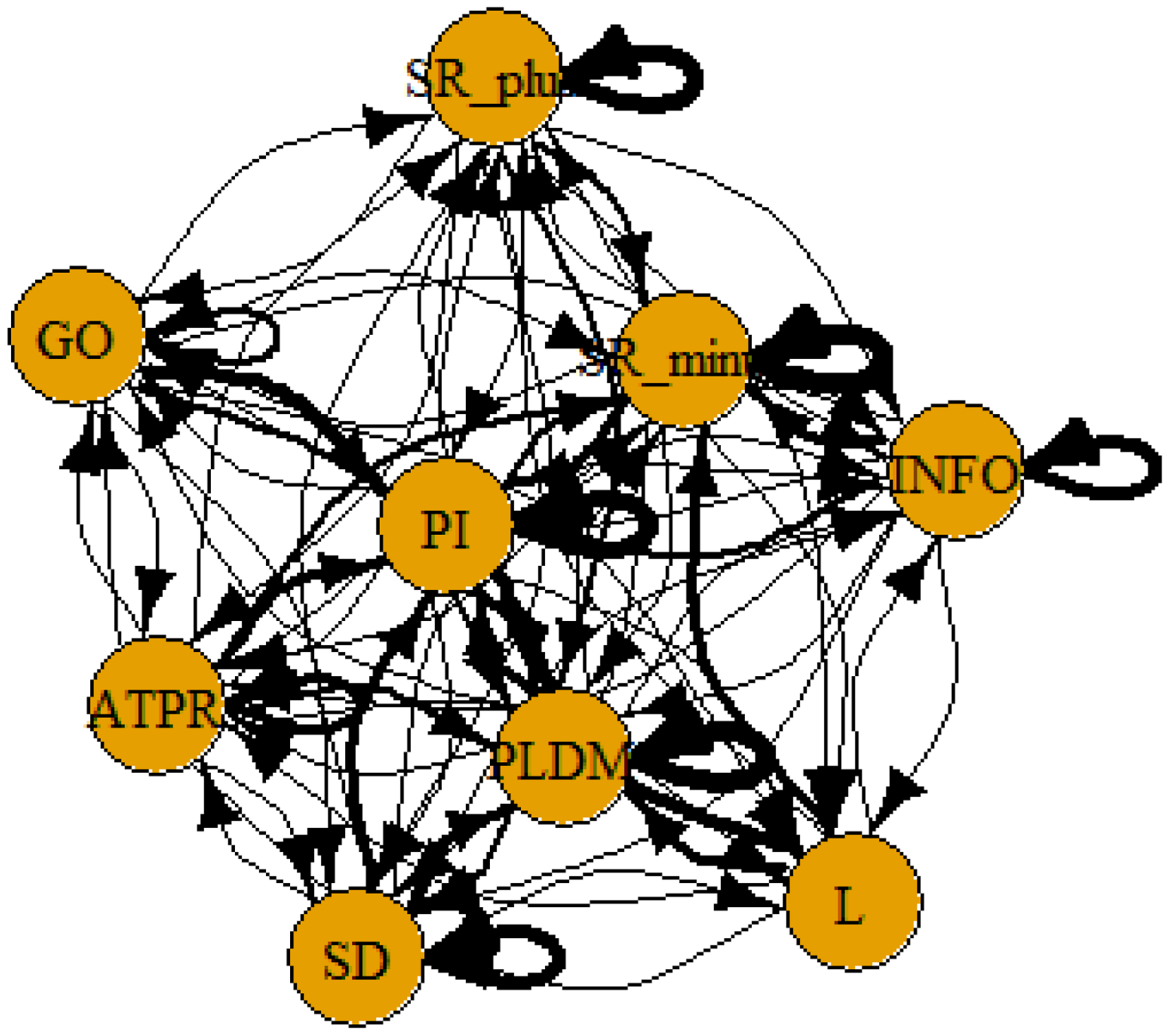
Brazil – COLDSTORE, all conditional probabilities.

**Figure 10:**
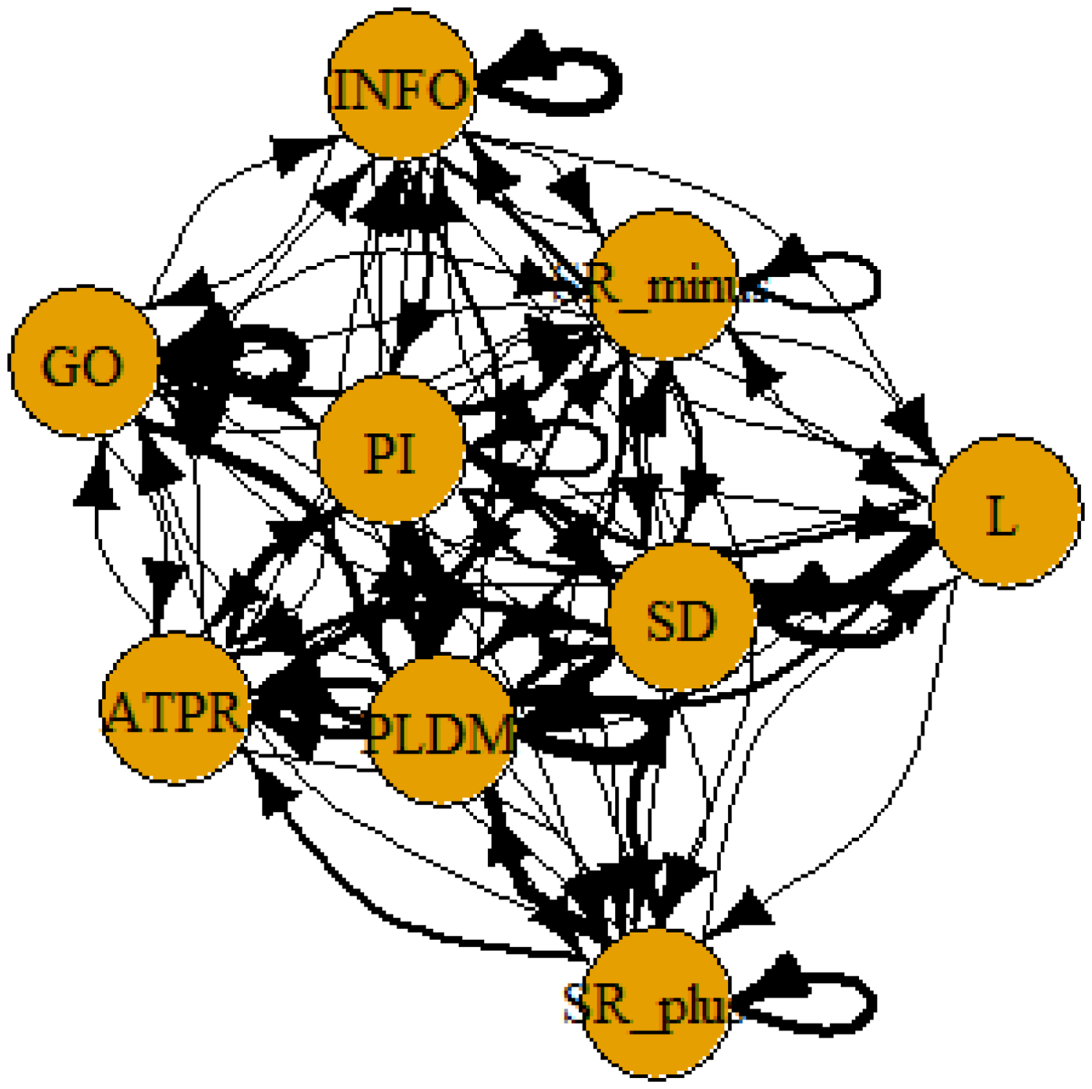
Germany – COLDSTORE, all conditional probabilities.

**Figure 11:**
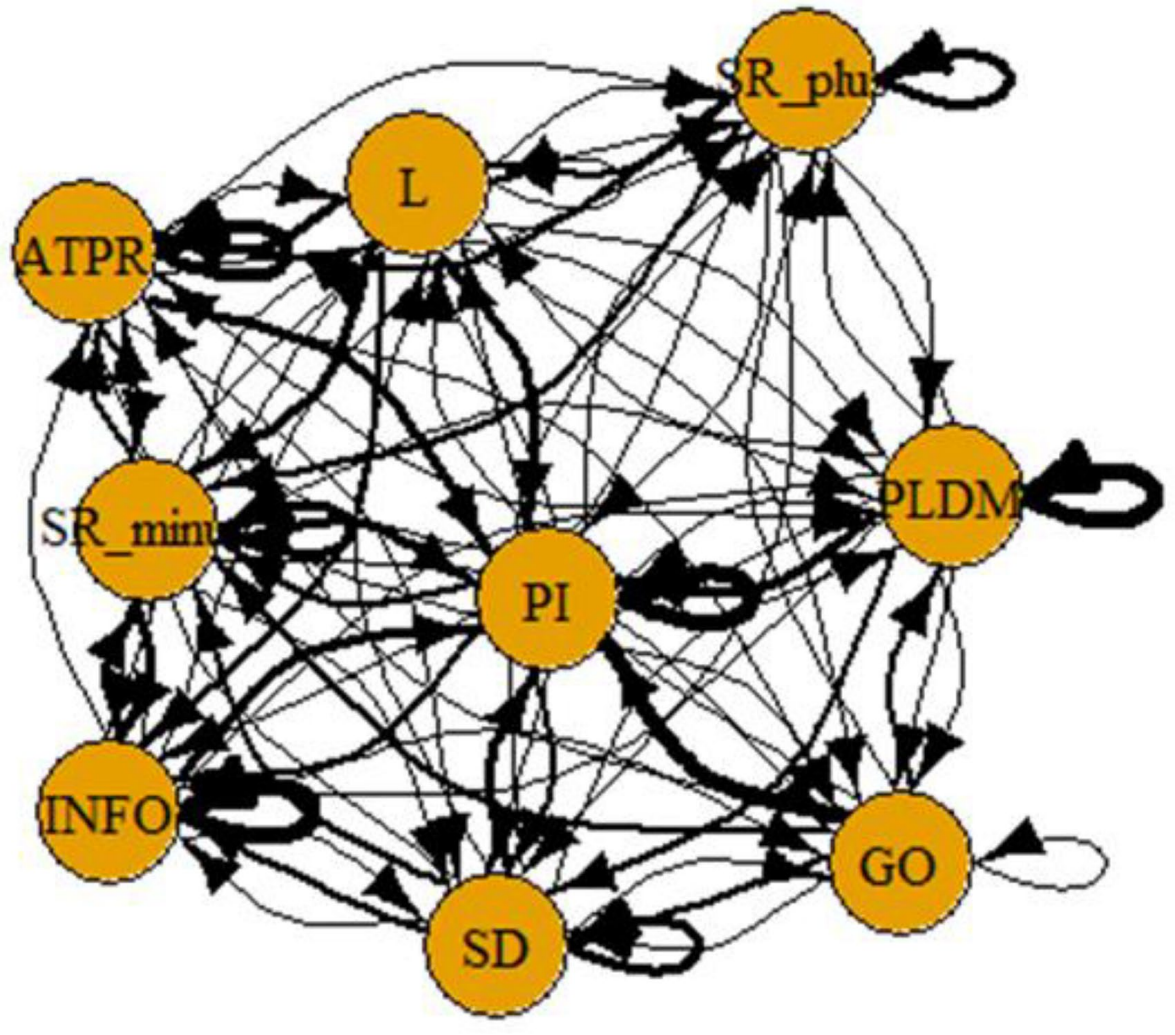
Philippines – COLDSTORE, all conditional probabilities.

**Figure 12:**
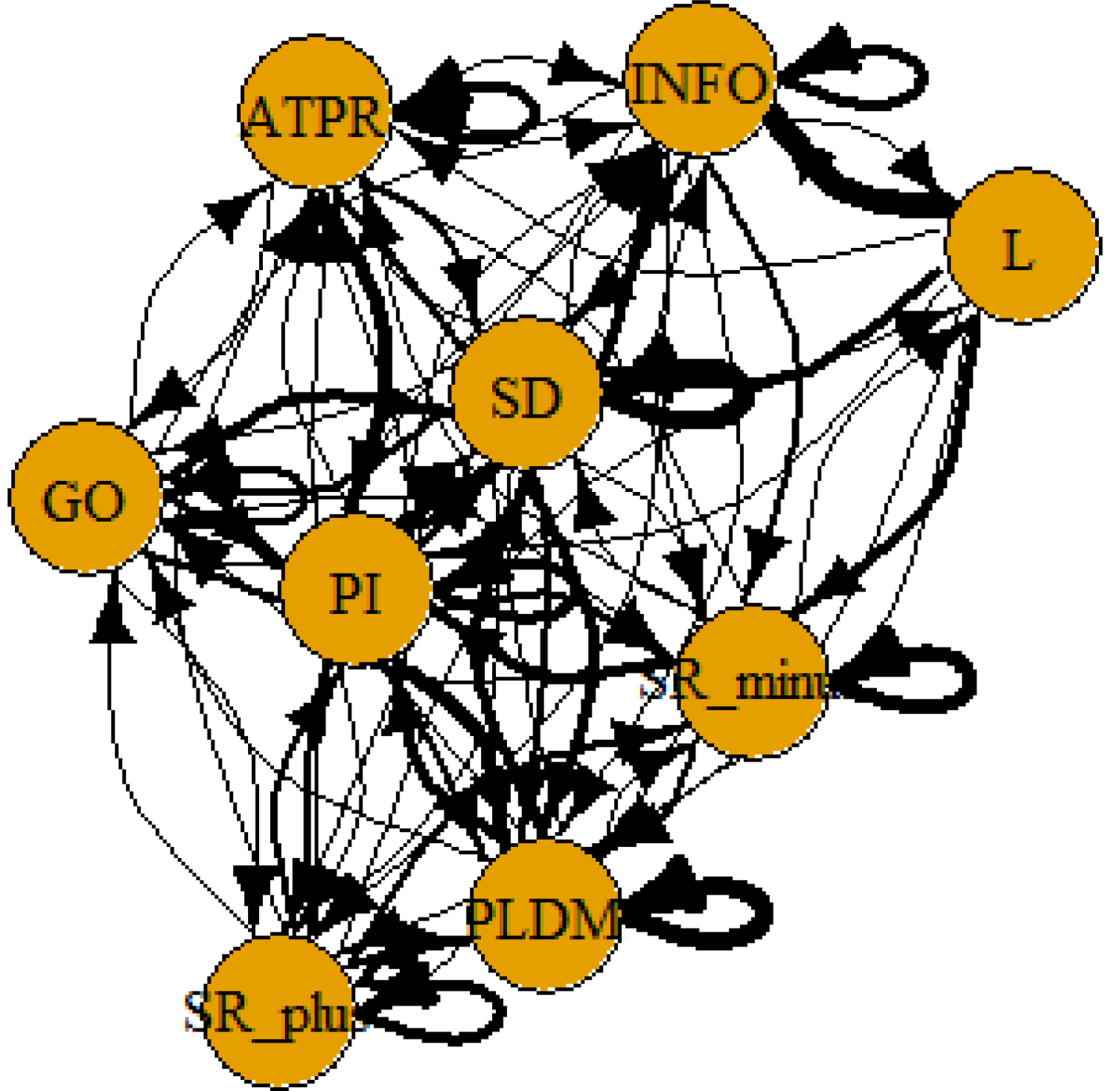
United States – COLDSTORE, all conditional probabilities.

**Figure 13:**
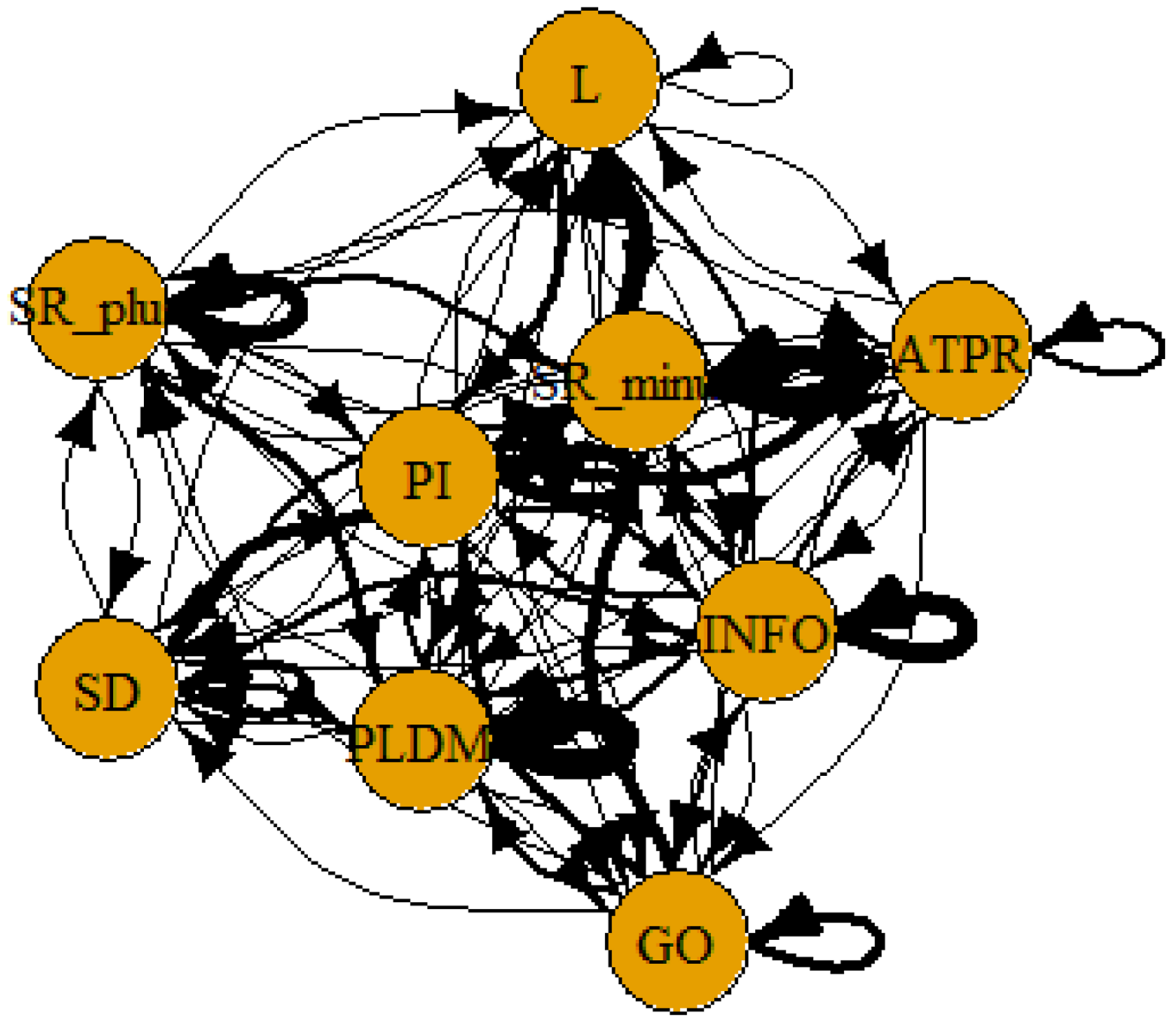
Brazil – WINFIRE, all conditional probabilities.

**Figure 14:**
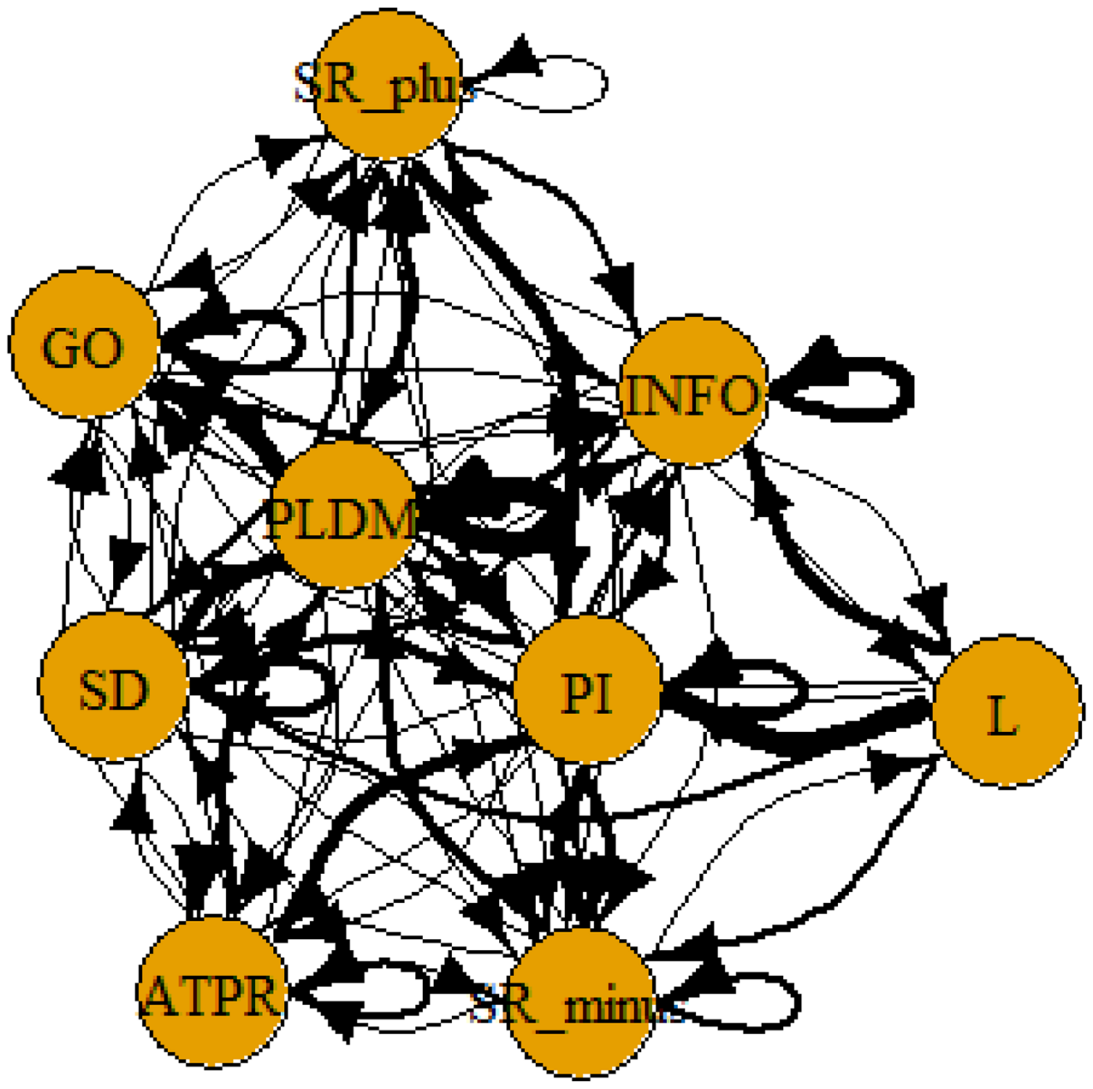
Germany – WINFIRE, all conditional probabilities.

**Figure 15:**
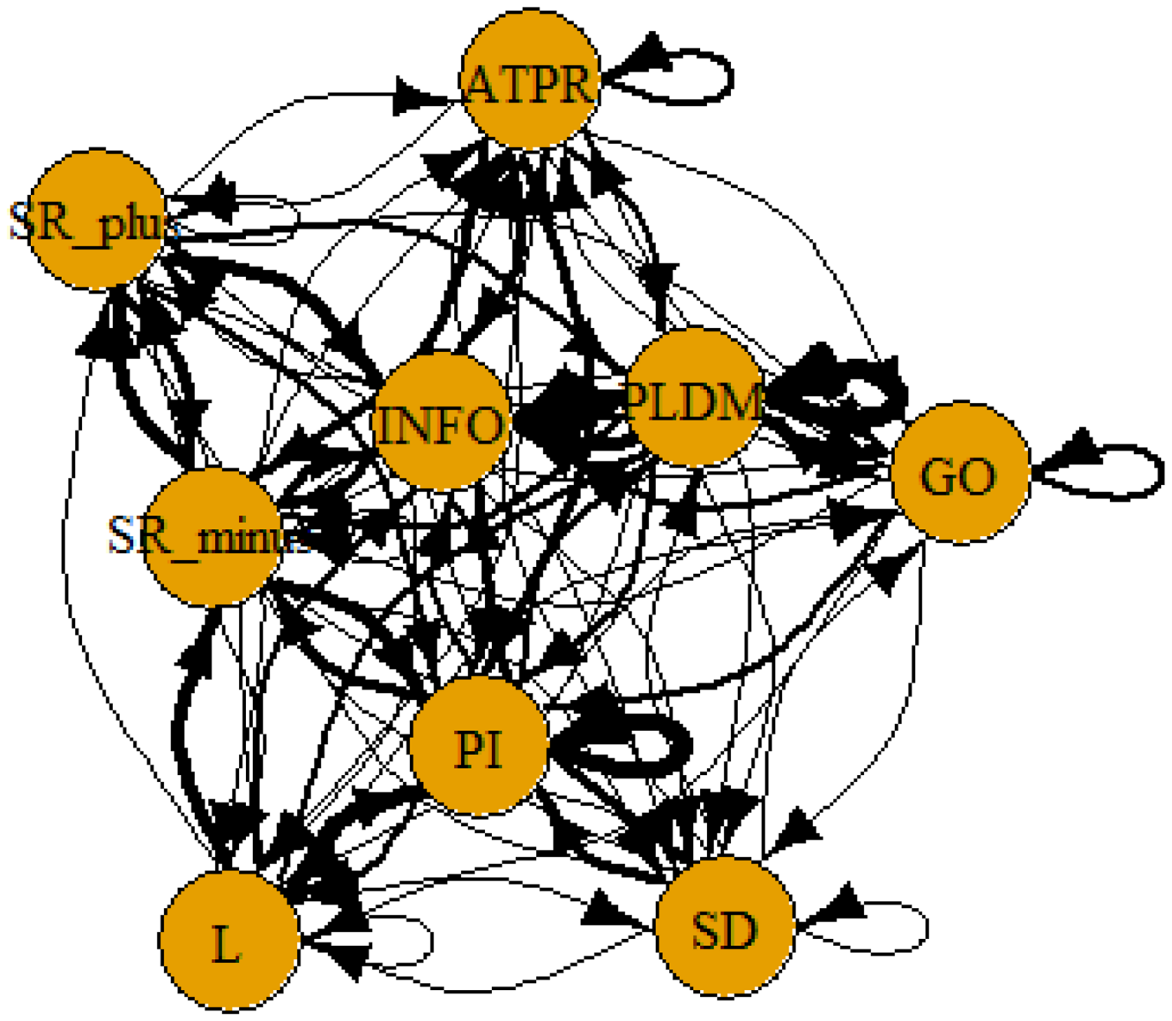
Philippines – WINFIRE, all conditional probabilities.

**Figure 16:**
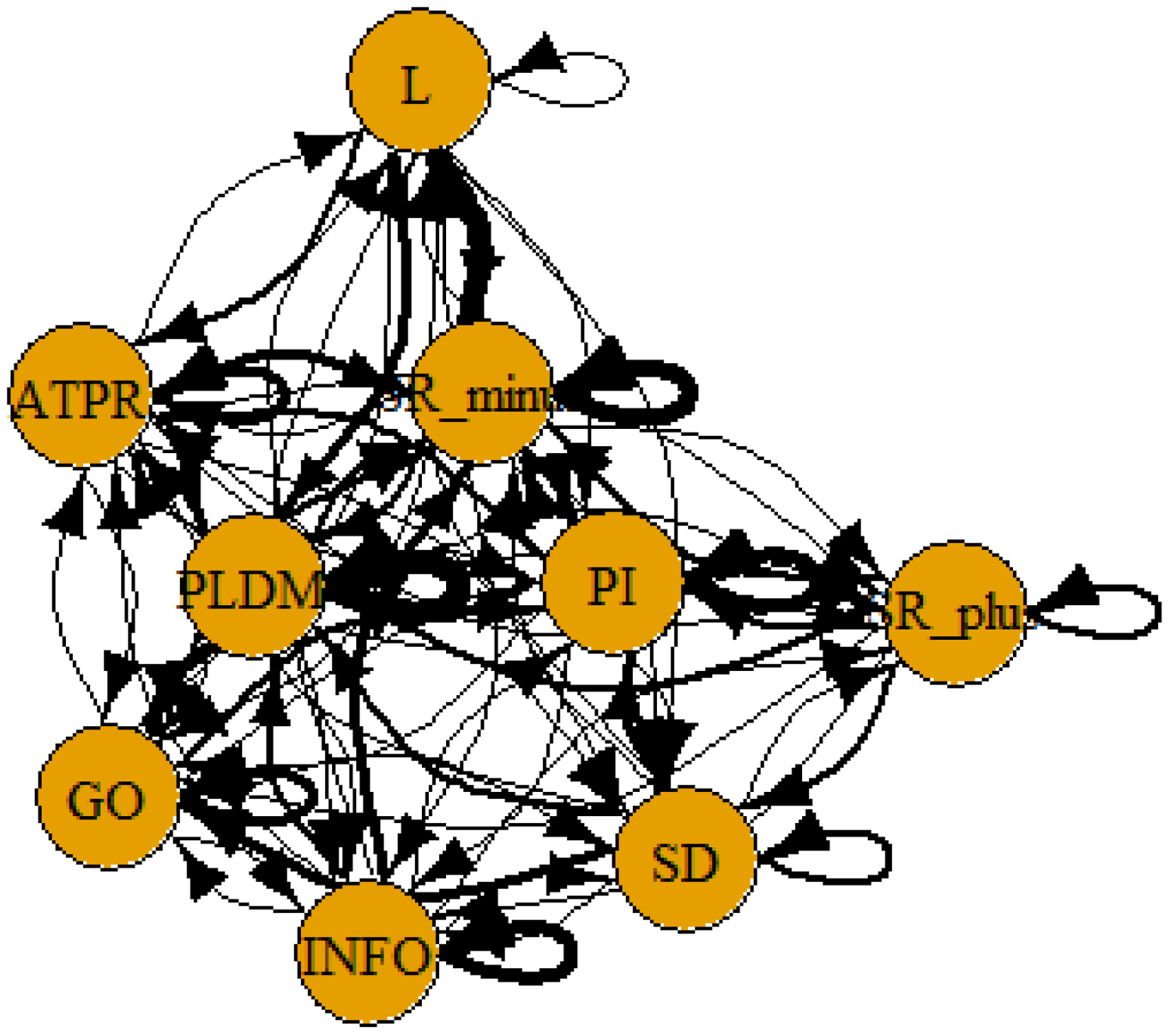
United States – WINFIRE, all conditional probabilities.

**Figure 17:**
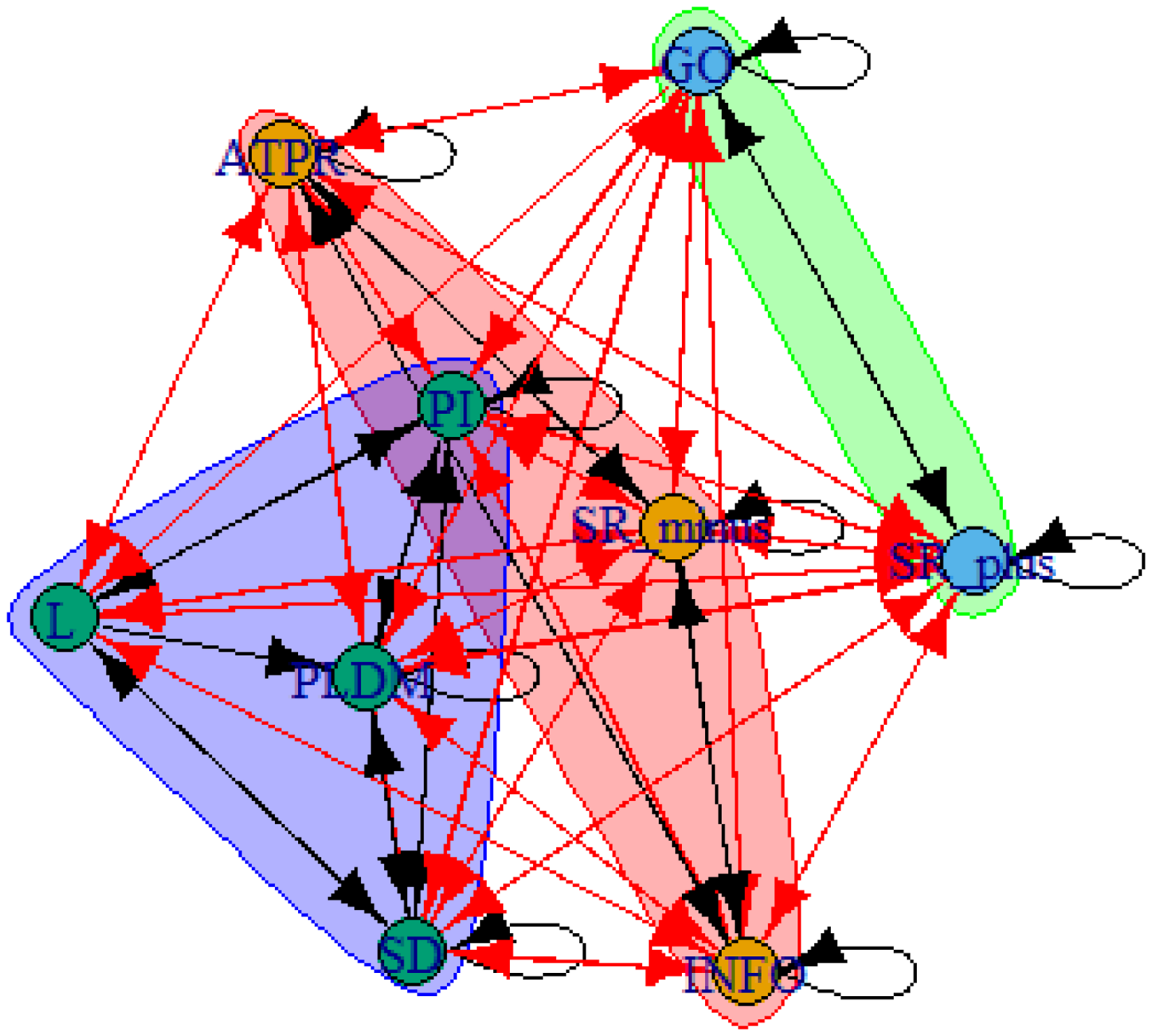
Brazil – COLDSTORE: Community clusters for transitional probabilities.

**Figure 18:**
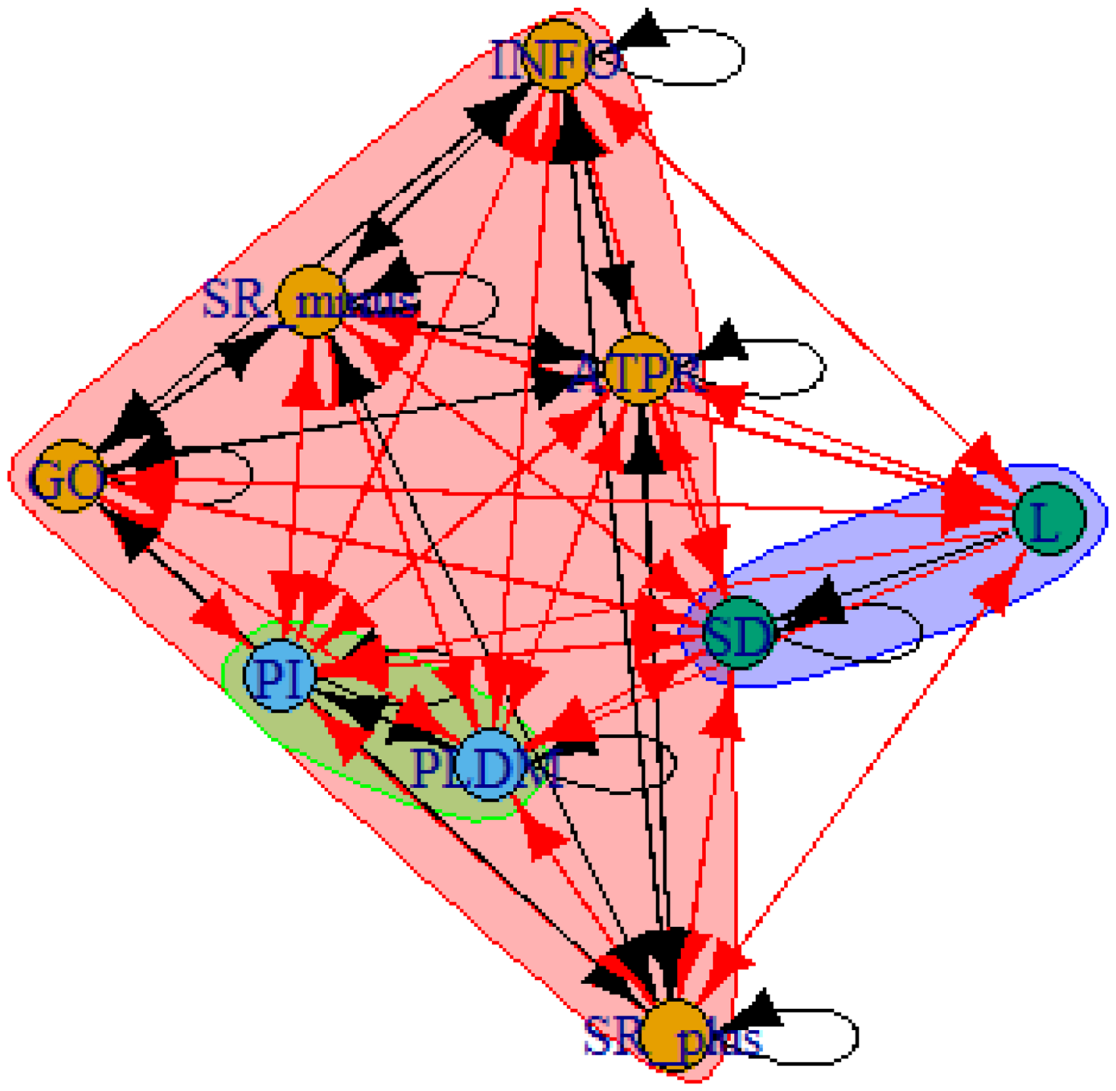
Germany – COLDSTORE: Community clusters for transitional probabilities.

**Figure 19:**
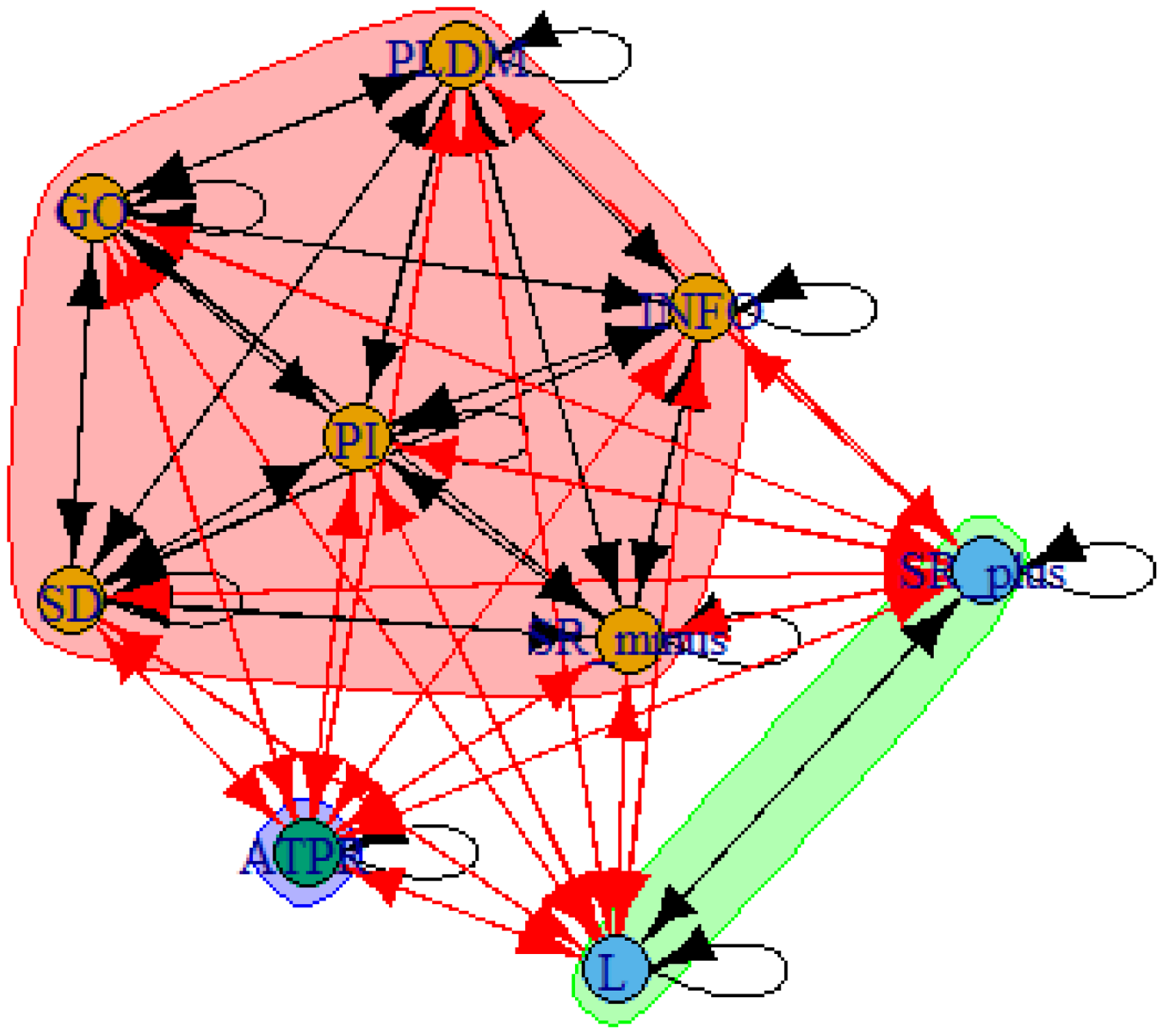
Philippines – COLDSTORE: Community clusters for transitional probabilities.

**Figure 20:**
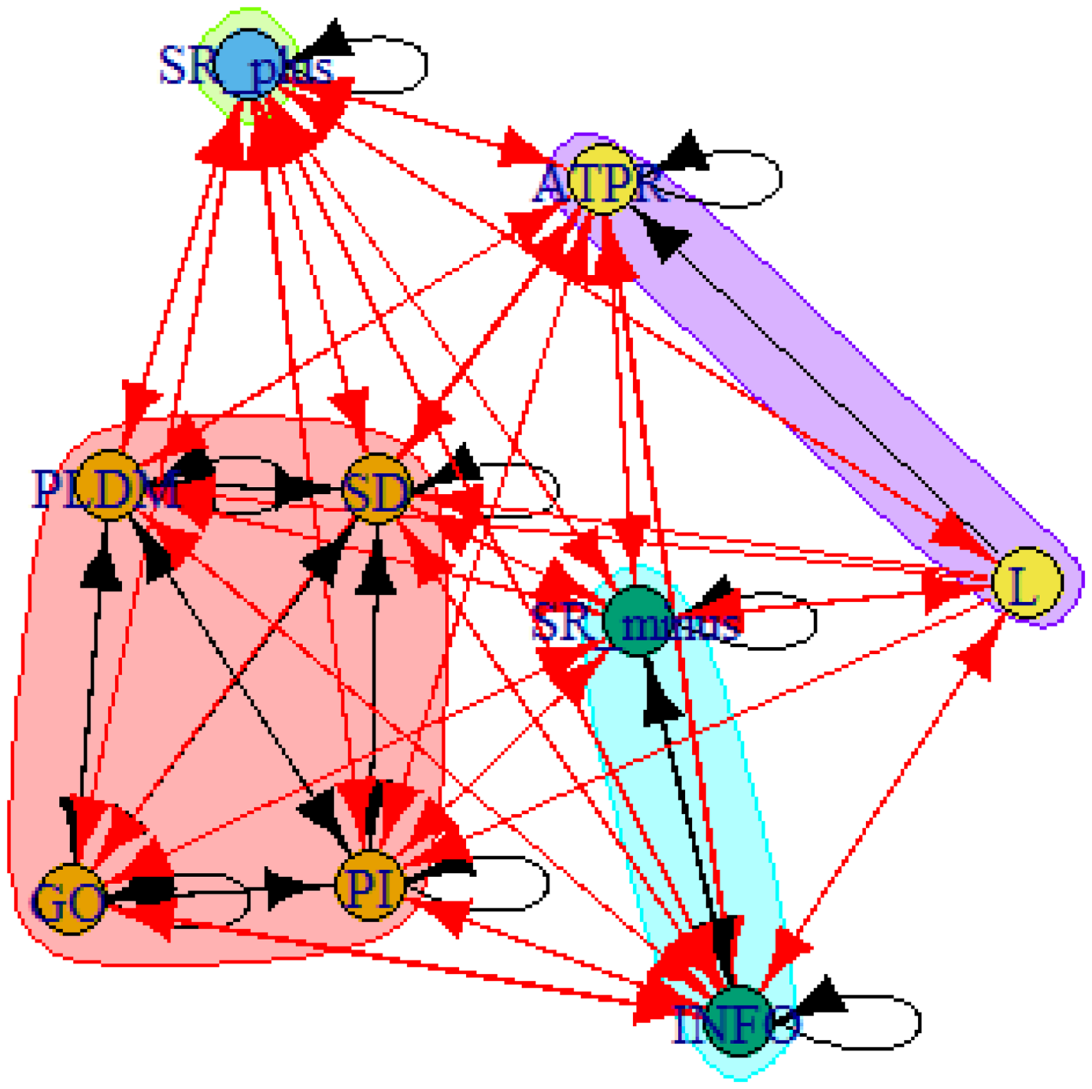
United States – COLDSTORE: Community clusters for transitional probabilities.

**Figure 21:**
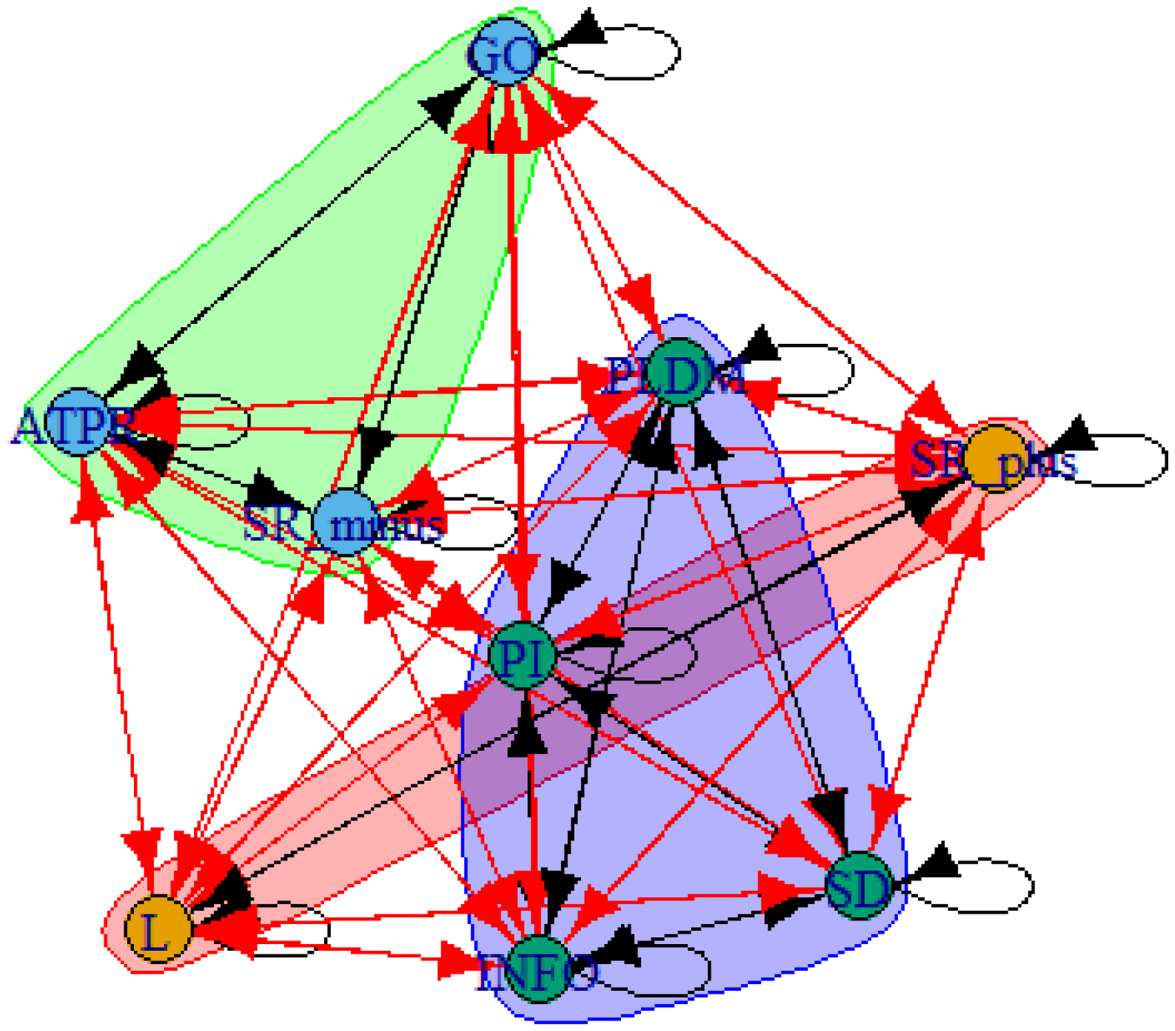
Brazil – WINFIRE: Community clusters for transitional probabilities.

**Figure 22:**
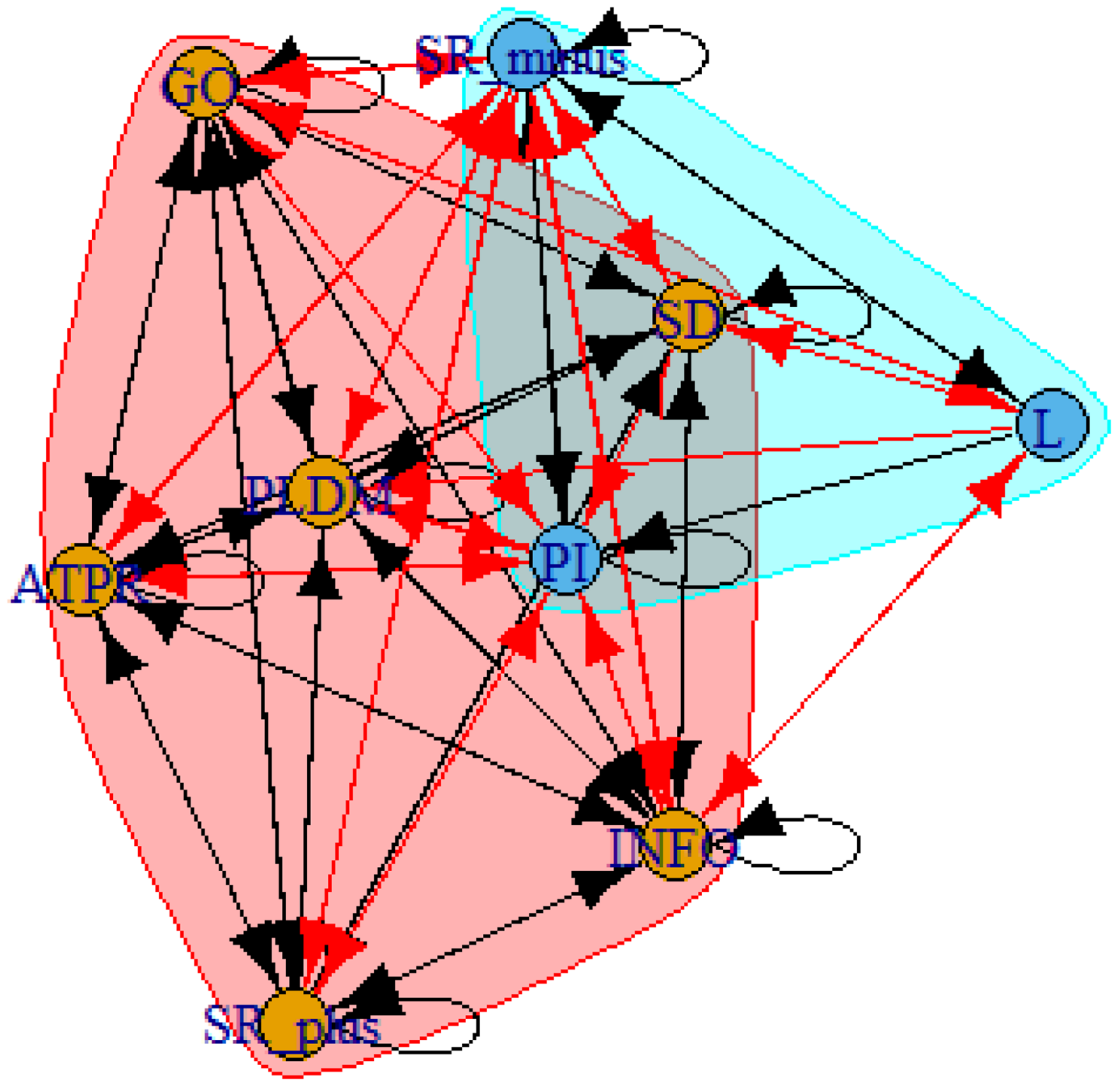
Germany – WINFIRE: Community clusters for transitional probabilities.

**Figure 23:**
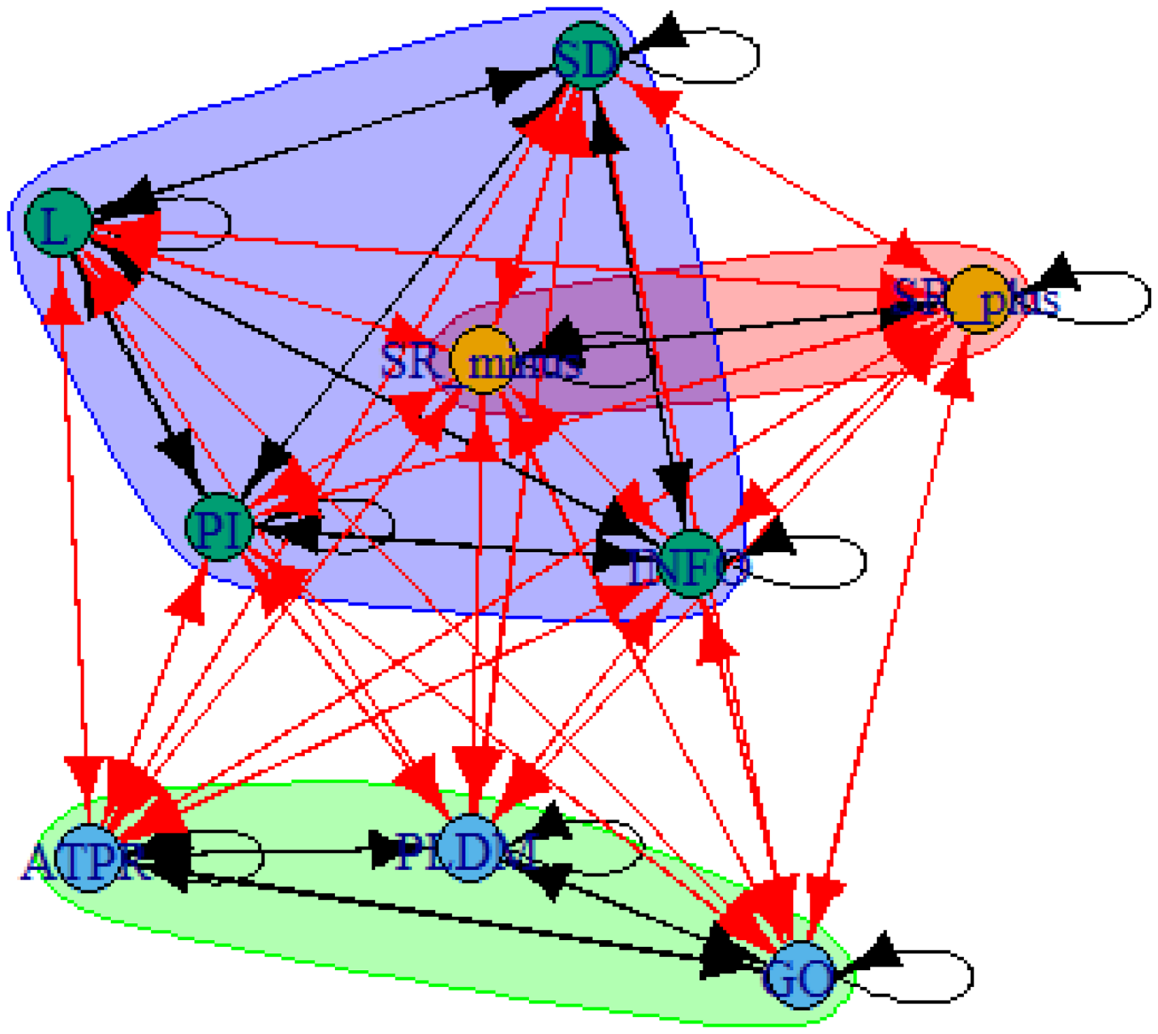
Philippines – WINFIRE: Community clusters for transitional probabilities.

**Figure 24:**
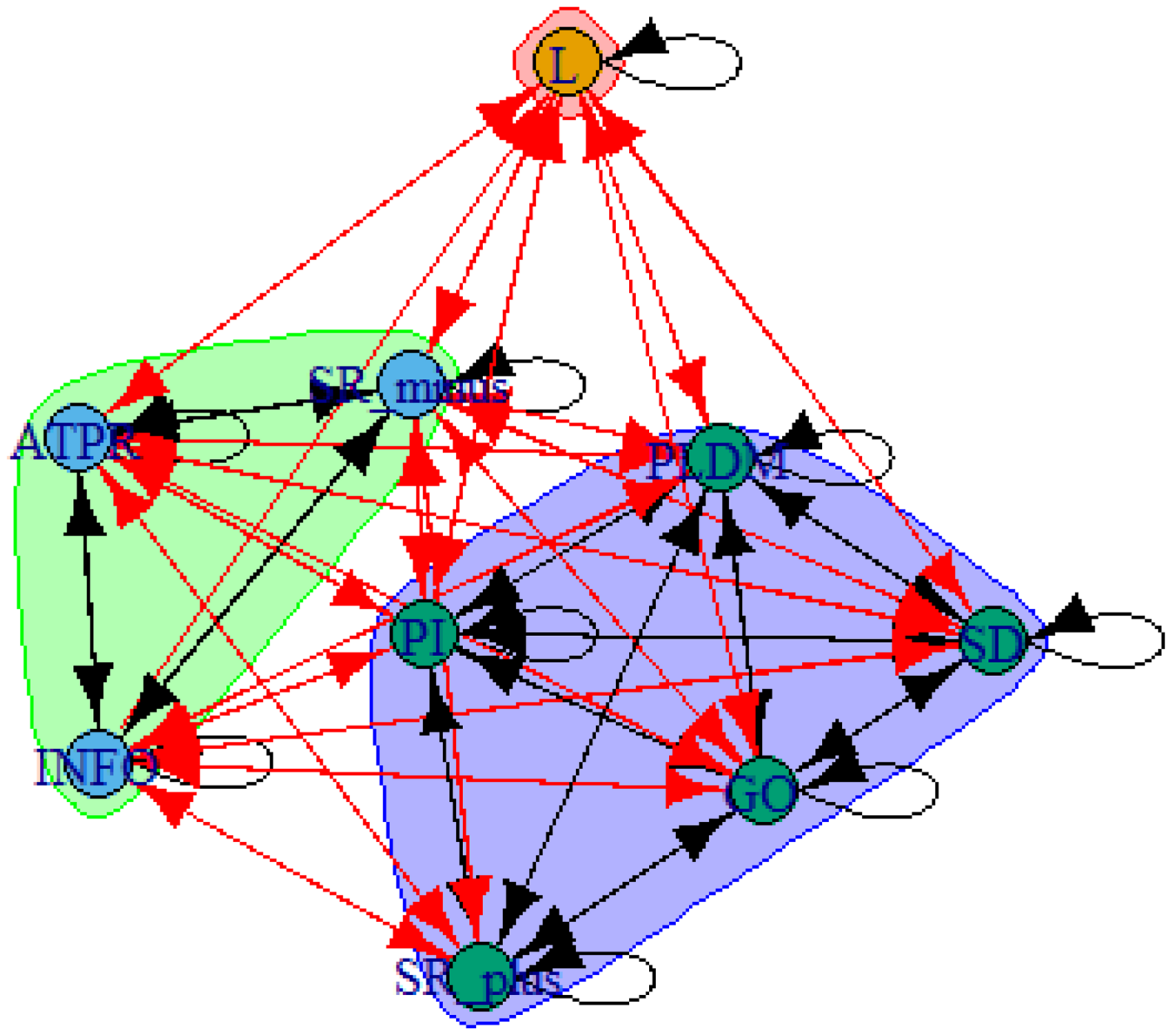
United States – WINFIRE: Community clusters for transitional probabilities.

We compared the percentages in the three most common transitions from Problem identification (PI) to Problem identification; from Problem identification to Negative self-reference (SR−); and from Problem identification to Planning, decision making, and action (PLDM) in WinFire. The percentages did not differ significantly among the four countries in WinFire, *χ*^2^ (6) = 6.27, *p* > 0.05, ns. For WinFire (see Table 1), Brazilians made primarily Problem identification (31%) and Negative self-reference (23%) statements after problem identification (PI). Germans had the most statement transitions regarding “Planning, decision making, and action” (21%) followed by Problem identification (20%) after PI. Filipinos showed primarily Problem identification (29%) and Negative self-reference (18%) statements after PI. US participants made primarily statements regarding Problem identification (26%) and Negative self-reference (25%) after PI.

**Table 1:** Highest average statement transition percentages stemming from problem identification for WINFIRE and COLDSTORE.

Country	WINFIRE	Statement transition percent	COLDSTORE	Statement transition percent
	Highest statement transition following Problem Identification		Highest statement transition following Problem Identification	
Brazil	Problem identification	31%	Problem identification	37%
	Negative self-reference	23%	Planning, decision making, and action	19%
	Planning, decision making, and action	14%	Negative self-reference	15%
Germany	Planning, decision making, and action	21%	Planning, decision making, and action	41%
	Problem identification	20%	Problem identification	15%
	Negative self-reference	18%	Situation description	12%
Philippines	Problem identification	29%	Problem identification	29%
	Negative self-reference	18%	Information gathering	13%
	Laughter	13%	Negative self-reference	13%
United States	Problem identification	26%	Planning, decision making, and action	26%
	Negative self-reference	25%	Problem identification	22%
	Planning, decision making, and action	17%	Negative self-reference	16%

We also compared the percentages in the three most common transitions from Problem identification (PI) to Problem identification; from Problem identification to Negative self-reference (SR−); and from Problem identification to Planning, decision making, and action (PLDM) in Coldstore. The percentages differed significantly among the four countries in Coldstore, *χ*^2^ (6) = 30.03, *p* < 0.001. For Coldstore (see Table 1), the most frequent problem-solving statements after problem identification (PI) for Brazilians were Problem identification (37%) and Planning, decision making, and action (19%). Germans made mostly Planning, decision making and action (41%) and Problem identification (15%) after PI. Filipinos made mostly Problem identification (29%) and Information gathering (13%) and Negative self-reference (13%) statements after PI. The US participants showed statements regarding Planning, decision making, and action (26%) and problem identification (22%) after PI.

We then compared the same three transition percentages between Winfire and Coldstore. They differed significantly between the two simulations, *χ*^2^ (2) = 13.82, *p* < 0.001. The transition to Problem identification was similar in both simulations, but more Negative self-reference (SR−) transitions were found in Winfire and more Planning, decision making, and action (PLDM) transitions were found in Coldstore.

Additionally, we run further analyses in the R-program on state-transitions of the complex problem-solving steps. For COLDSTORE, Brazilian, German, and Filipino data showed each 3 community clusters, and US data showed 4 community clusters. For all countries, the two steps Problem identification (PI) and Planning, decision making, and action (PLDM) were always in the same cluster. COLDSTORE data from all four countries have in common that negative self-reflections and emotions (SR−) were always in the same cluster as information gathering (INFO).

For WINFIRE, Brazilian, Filipino, and US data showed 3 community clusters, German data showed 2 community clusters. Only for the Brazilian and US samples, the two steps PI and PLDM were in the same cluster. For WINFIRE, SR− was only in the same cluster as INFO in one sample, the US sample.

The clusters containing PI varied among cultures. For the German sample, for example, only one other step in COLDSTORE and two other steps in WINFIRE were in the same cluster as PI. For the US sample, 3 other steps in COLDSTORE and 4 other steps in WINFIRE were in the same cluster as PI.

From a cognitive perspective, it is surprising that negative emotions and self-references (SR−) were always central among the problem-solving clusters. In complex situations, negative emotions seem to provide situational impressions that can then motivate and lead to modifications in the problem-solving approach.

## Discussion

The purpose of this study was to examine the process of complex problem-solving and cultural differences among Brazil, Germany, the Philippines, and the United States. Our hypotheses included, Brazilians making primarily emotional and self-related statements, Germans engaging primarily in planning, Filipinos gathering additional information, and Americans stating solutions.

The first hypothesis regarding Brazil was partially supported. Data showed 23% of Brazilians making negative self-references (SR−) following problem identification (PI) in WinFire. These findings are partially consistent with previous research showing the high emotional expressiveness of Brazilians ranking number 10 out of 32 countries (Matsumoto, 2008). However, in both WinFire and Coldstore, Brazilians continue to elaborate on the problem situation with 37% of Brazilians stating problem identification statements (PI) after problem identifications in Coldstore and 31% in WinFire. Thus, elaborating on the problem is the most frequent transition.

The second hypothesis regarding German participants was supported; 21% of German participants responded to problem identification with planning, decision making and action (PLDM) in WinFire, and 41% of German participants responded with planning, decision making, and action (PLDM) to problem identification (PI) in Coldstore. These results are consistent with previous research showing the importance of planning in the German culture (e.g., Strohschneider and Güss, 1998; Hofstede, 2001). Additionally, these results reflect Germans’ desire for thinking, planning ahead, and taking action in the opportunities given (Tipandjan et al., 2012).

The third hypothesis regarding Filipino participants was partially supported, which showed 29% of Filipinos in WinFire and 29% of Filipinos in Coldstore were involved in problem identification (PI) after problem identifications and 13% made statements related to information gathering (INFO) in Coldstore. Problem identification is a part of gathering further information; one elaborates on the problem, further analyzes it, and tries to understand it. It shows a more cautious approach to problem solving. These results are consistent with the analysis of dynamic decisions (and not thinking aloud) in Coldstore where Filipinos, in comparison to the participants from the other countries, showed the most cautious approach (Güss and Dörner, 2011). These results are also supported by Builtjens and Noorderhaven (1996) who showed that the cautious decision-making process is partially due to a culture which is highly context dependent.

The fourth hypothesis regarding US Americans was only supported for Coldstore which showed 26% of American participants involved in planning, decision making, and action (PLDM) after identifying a problem. Further problem identification played an important role in WinFire with 26% and in Coldstore with 22% of the statements following problem identification (PI). The results reveal a need to understand the problem situation better, before making decisions. Other research reveals Americans being very conscious about the decision-making process as well as making choices depending on the information that is given instead of the loyalty of a company or brand (Leng and Botelho, 2010).

Summarizing the results focusing on the simulation, we find for WinFire, that in three of the four countries (Brazil, Philippines, United States), the primary reactions to recognizing a problem were problem-related statements and negative self-references, i.e., negative emotions and negative statements regarding one’s self-wroth (SR−). Only German participants showed primarily planning and decision making (PLDM) after problem identification (PI). The countries did not differ significantly in their transition percentages.

For Coldstore, the countries differed significantly in their transition percentages. The most frequent problem-solving statements after problem identification (PI) in three of the four countries (Brazil, Germany, United States) were planning, decision making, and action (PLDM); and problem identification – in different order though. Filipino participants also made statements regarding problem identification, but also regarding information gathering (INFO) and negative self-references (SR−).

Two general findings are worth mentioning contradicting our predictions. First, in both situations, the most frequent statements following problem identification (PI) are further problem identification statements (PI), negative self-references (SR−), and planning, decision making, and action (PLDM). Most participants, regardless of their cultural background, further elaborate on the problem situation, but then show either negative emotions such as stress or frustration or show self-derogatory statements like “I will never be a good fire fighter” or “I am not good at this.” before they go on with further planning and decision making. Contradicting the rational “homo economicus” decision-making model, people are affected by complex and dynamic problems: they often react overwhelmed, stressed, and their feeling of competence is attacked. Emotions, motivations, and cognition interact when people deal with complex problems (see Dörner and Güss, 2013; Dörner and Güss, 2022).

Second, there were no cultural differences in WinFire regarding transitions after problem identification (PI). In Coldstore, however, we found significant differences and see more cultural differences in the transitions after problem identification (PI). One possible explanation is related to the transparency and uncertainty related to the two simulations. In WinFire, it is obvious when a fire starts, where it starts, and if it is an imminent threat to a city or not. It is obvious how distant trucks and helicopters are from the fires. Wind strength and direction are also shown. Thus, even though the problem is highly dynamic, it is relatively transparent. Coldstore, however, is more uncertain. It is unclear why the temperature oscillates, and why the temperature does not seem to react to the changes on the control wheel. This uncertainty leaves room for interpretation and for discovery and thus allows the cultural decision-making strategies to shine. This discussion about the different task demands of WinFire and Coldstore is also reflected in the significant differences of the transition probabilities between the two simulations in the three most frequent steps (from Problem identification PI to Problem identification PI, to Negative self-reference SR−, and to Planning, decision making, and action PLDM.) The transition to Problem identification was similar in both simulations, but more Negative self-reference transitions (SR−) were found in Winfire and more Planning, decision making, and action (PLDM) transitions were found in Coldstore.

Our study also has limitations. The first limitation is within-country diversity. It is apparent that within every culture there also exist various subcultures and we only include two student samples from different cities in each country. Another limitation is related to the think-aloud method. For some participants, it was unusual and uncomfortable to talk aloud, especially for the Filipino participants (see also differences in think-aloud between European and Asian Americans, Kim, 2002; on language and reasoning see also Papafragou et al., 2007). Future research could, for example, consider group-versus individual-related differences in complex problem solving across various cultural regions. A third limitation is related to the spinglass community detection method in igraph, as items (transition frequencies in our case) deterministically belong to one community only.

In sum, this study showed an in-depth log-linear analysis of the complex and dynamic problem-solving strategies in four countries during two different simulated tasks. Findings showed unique cultural preferences in strategies when identifying problems when the task was uncertain, but not when the task was relatively transparent. It is the cultural upbringing in our mind that triggers preferred ways in dealing with uncertain problem situations.

## Data availability statement

The original contributions presented in the study are included in the article. Data for this study can be found in the Supplementary material section. Further inquiries can be directed to the corresponding author.

## Ethics statement

The studies involving human participants were reviewed and approved by The University of North Florida Institutional Review Board together with the ethics committees in every country – where required. The participants provided their written informed consent to participate in this study.

## Author contributions

WS and JH were primarily analyzing the transition data and creating the figures. CG was conducting the R-analyses, creating the figures in R, and writing most parts of the manuscript. All authors contributed to the article and approved the submitted version.

## Funding

This research was supported in part by the National Science Foundation grant #0218203 to the last author and by the University of North Florida’s John A. Delaney Presidential Professorship to the last author.

## Conflict of interest

The authors declare that the research was conducted in the absence of any commercial or financial relationships that could be construed as a potential conflict of interest.

## Publisher’s note

All claims expressed in this article are solely those of the authors and do not necessarily represent those of their affiliated organizations, or those of the publisher, the editors and the reviewers. Any product that may be evaluated in this article, or claim that may be made by its manufacturer, is not guaranteed or endorsed by the publisher.
