# Supplementary material for: “Oh no, the forest is burning!” cultural differences in the complex problem-solving process only under high uncertainty
Source: Front Psychol. 2022 Dec 22;13:965623. doi: 10.3389/fpsyg.2022.965623 (PMC9815707; doi:10.3389/fpsyg.2022.965623)
Supplement: Supplementary file 1 [file Data_Sheet_1.docx]

Appendix 1

*Coding System for the 10 General Steps for the Think-Aloud Analyses*

| Complex problem- solving step | Abbre-viation | Definition of category | Example from WINFIRE | Example from COLDSTORE |
| --- | --- | --- | --- | --- |
| Situation description | SD | Describing or summarizing positive or neutral aspects of the situation | “The truck is close to city 3” | “The temperature is almost ideal” |
| Problem identification | PI | Identifying and expressing a problem or negative aspect of a situation | “There is a fire in the forest!” | “Products are freezing.” |
| Formulation of goals | GO | Stating a specific goal or strategy | “I want to extinguish fires.” | “I want to keep the temperature stable.” |
| Gathering of information | INFO | Expressing need for information by asking question to oneself or facilitator | “Let’s see how much water truck 4 has.” | “Why is the temperature always changing?” |
| Attributions and predictions | ATPred | Stating possible causes for certain events or possible developments | “… because the truck is so slow.” | “… because I switched it so much.” |
| Planning, decision making, and action | PLDM | Expressing plan, solution, or justification for a specific solution | “Truck 5 will clear the area next to city 2.” | “I will try to move the control wheel in decrements of 5.” |
| Positive self-evaluations and emotions | SR+ | Positive evaluation of effectiveness of solution as expressed in “I” statements and positive emotions | “I am getting a handle of this.” | “This is fun!” |
| Negative self-evaluations and emotions | SR- | Negative evaluation of effectiveness of solution as expressed in “I” statements and negative emotions | “I am bad at this.” | “This is stressful.” |
| Laughing | L | laughing | hahaha | hehehe |
| Other | O | Repeating the exact same statement; every reply statement of the participant after a comment of the experimenter; not understandable statements | “mmmhhh” | “ahhhh” |
