# Supplementary material for: “Oh no, the forest is burning!” cultural differences in the complex problem-solving process only under high uncertainty
Source: Front Psychol. 2022 Dec 22;13:965623. doi: 10.3389/fpsyg.2022.965623 (PMC9815707; doi:10.3389/fpsyg.2022.965623)
Supplement: Supplementary file 2 [file Data_Sheet_2.docx]

Table 1

*Brazil WINFIRE Contingency Table*

| OBSERVED FREQUENCIES | | |  |  |  |  |  |  |  |  |
| --- | --- | --- | --- | --- | --- | --- | --- | --- | --- | --- |
|  | SD | PI | GO | INFO | ATPRED | PLANDM | SR- | SR+ | L | TOTAL |
| SD | 33 | 48 | 6 | 29 | 9 | 30 | 25 | 7 | 4 | 191 |
| PI | 33 | 254 | 25 | 102 | 42 | 117 | 185 | 18 | 35 | 811 |
| GO | 8 | 35 | 36 | 13 | 3 | 26 | 41 | 7 | 1 | 170 |
| INFO | 16 | 70 | 15 | 204 | 8 | 75 | 119 | 15 | 20 | 542 |
| ATPRED | 4 | 44 | 6 | 14 | 28 | 28 | 41 | 3 | 12 | 180 |
| PLANDM | 39 | 137 | 26 | 70 | 39 | 440 | 88 | 27 | 11 | 877 |
| SR- | 26 | 202 | 37 | 114 | 39 | 101 | 394 | 28 | 33 | 974 |
| SR+ | 8 | 16 | 5 | 18 | 5 | 30 | 26 | 57 | 6 | 171 |
| L | 8 | 24 | 2 | 18 | 12 | 10 | 51 | 4 | 1 | 130 |
| TOTAL | 175 | 830 | 158 | 582 | 185 | 857 | 970 | 166 | 123 | 4046 |
|  |  |  |  |  |  |  |  |  |  |  |
| CONDITIONAL PROBABLITIES | | | |  |  |  |  |  |  |  |
|  | SD | PI | GO | INFO | ATPRED | PLANDM | SR- | SR+ | L | TOTAL |
| SD | 0.17 | 0.25 | 0.03 | 0.15 | 0.05 | 0.16 | 0.13 | 0.04 | 0.02 | 1 |
| PI | 0.04 | 0.31 | 0.03 | 0.13 | 0.05 | 0.14 | 0.23 | 0.02 | 0.04 | 1 |
| GO | 0.05 | 0.21 | 0.21 | 0.08 | 0.02 | 0.15 | 0.24 | 0.04 | 0.01 | 1 |
| INFO | 0.03 | 0.13 | 0.03 | 0.38 | 0.01 | 0.14 | 0.22 | 0.03 | 0.04 | 1 |
| ATPRED | 0.02 | 0.24 | 0.03 | 0.08 | 0.16 | 0.16 | 0.23 | 0.02 | 0.07 | 1 |
| PLANDM | 0.04 | 0.16 | 0.03 | 0.08 | 0.04 | 0.50 | 0.10 | 0.03 | 0.01 | 1 |
| SR- | 0.03 | 0.21 | 0.04 | 0.12 | 0.04 | 0.10 | 0.40 | 0.03 | 0.03 | 1 |
| SR+ | 0.05 | 0.09 | 0.03 | 0.11 | 0.03 | 0.18 | 0.15 | 0.33 | 0.04 | 1 |
| L | 0.06 | 0.18 | 0.02 | 0.14 | 0.09 | 0.08 | 0.39 | 0.03 | 0.01 | 1 |
| TOTAL | 0.04 | 0.21 | 0.04 | 0.14 | 0.05 | 0.21 | 0.24 | 0.04 | 0.03 | 1 |
|  |  |  |  |  |  |  |  |  |  |  |
|  |  |  |  |  |  |  |  |  |  |  |
| EXPECTED FREQUENCIES | | |  |  |  |  |  |  |  |  |
|  | SD | PI | GO | INFO | ATPRED | PLANDM | SR- | SR+ | L | TOTAL |
| SD | 8.26 | 39.18 | 7.46 | 27.47 | 8.73 | 40.46 | 45.79 | 7.84 | 5.81 | 191 |
| PI | 35.08 | 166.37 | 31.67 | 116.66 | 37.08 | 171.78 | 194.43 | 33.27 | 24.65 | 811 |
| GO | 7.35 | 34.87 | 6.64 | 24.45 | 7.77 | 36.01 | 40.76 | 6.97 | 5.17 | 170 |
| INFO | 23.44 | 111.19 | 21.17 | 77.96 | 24.78 | 114.80 | 129.94 | 22.24 | 16.48 | 542 |
| ATPRED | 7.79 | 36.93 | 7.03 | 25.89 | 8.23 | 38.13 | 43.15 | 7.39 | 5.47 | 180 |
| PLANDM | 37.93 | 179.91 | 34.25 | 126.15 | 40.10 | 185.76 | 210.25 | 35.98 | 26.66 | 877 |
| SR- | 42.13 | 199.81 | 38.04 | 140.11 | 44.54 | 206.31 | 233.51 | 39.96 | 29.61 | 974 |
| SR+ | 7.40 | 35.08 | 6.68 | 24.60 | 7.82 | 36.22 | 41.00 | 7.02 | 5.20 | 171 |
| L | 5.62 | 26.67 | 5.08 | 18.70 | 5.94 | 27.54 | 31.17 | 5.33 | 3.95 | 130 |
| TOTAL | 175 | 830 | 158 | 582 | 185 | 857 | 970 | 166 | 123 | 4046 |
|  |  |  |  |  |  |  |  |  |  |  |
|  |  |  |  |  |  |  |  |  |  |  |
| z-scores |  |  |  |  |  |  |  |  |  |  |
|  | SD | PI | GO | INFO | ATPRED | PLANDM | SR- | SR+ | L |  |
| SD | 9.01 | 1.62 | -0.56 | 0.32 | 0.09 | -1.90 | -3.61 | -0.31 | -0.78 |  |
| PI | -0.40 | 8.52 | -1.35 | -1.64 | 0.92 | -5.27 | -0.87 | -3.02 | 2.37 |  |
| GO | 0.25 | 0.02 | 11.88 | -2.56 | -1.79 | -1.92 | 0.04 | 0.01 | -1.90 |  |
| INFO | -1.69 | -4.71 | -1.47 | 16.58 | -3.71 | -4.50 | -1.18 | -1.68 | 0.95 |  |
| ATPRED | -1.42 | 1.34 | -0.41 | -2.58 | 7.22 | -1.89 | -0.38 | -1.69 | 2.90 |  |
| PLANDM | 0.20 | -4.05 | -1.62 | -6.11 | -0.20 | 23.74 | -10.93 | -1.73 | -3.48 |  |
| SR- | -2.92 | 0.20 | -0.20 | -2.74 | -0.97 | -9.48 | 13.82 | -2.22 | 0.73 |  |
| SR+ | 0.23 | -3.69 | -0.68 | -1.47 | -1.05 | -1.19 | -2.74 | 19.69 | 0.36 |  |
| L | 1.04 | -0.59 | -1.42 | -0.18 | 2.58 | -3.83 | 4.14 | -0.60 | -1.53 |  |

Table 2

*Brazil COLSTORE Contingency Table*

| OBSERVED FREQUENCIES | | |  |  |  |  |  |  |  |  |
| --- | --- | --- | --- | --- | --- | --- | --- | --- | --- | --- |
|  | SD | PI | GO | INFO | ATPRED | PLANDM | SR- | SR+ | L | **TOTAL** |
| SD | 161 | 112 | 21 | 26 | 32 | 128 | 35 | 28 | 4 | **547** |
| PI | 70 | 391 | 41 | 64 | 74 | 202 | 161 | 40 | 23 | **1066** |
| GO | 17 | 40 | 23 | 5 | 16 | 28 | 19 | 9 | 2 | **159** |
| INFO | 24 | 53 | 6 | 105 | 16 | 42 | 77 | 16 | 8 | **347** |
| ATPRED | 34 | 70 | 15 | 11 | 62 | 42 | 43 | 11 | 10 | **298** |
| PLANDM | 181 | 201 | 16 | 49 | 46 | 459 | 91 | 31 | 3 | **1077** |
| SR- | 24 | 180 | 22 | 72 | 49 | 73 | 377 | 45 | 26 | **868** |
| SR+ | 20 | 33 | 7 | 22 | 12 | 40 | 54 | 108 | 5 | **301** |
| L | 7 | 24 | 0 | 5 | 8 | 15 | 18 | 2 | 0 | **79** |
| TOTAL | 538 | 1104 | 151 | 359 | 315 | 1029 | 875 | 290 | 81 | **4742** |
|  |  |  |  |  |  |  |  |  |  |  |
| CONDITIONAL PROBABLITIES | | |  |  |  |  |  |  |  |  |
|  | SD | PI | GO | INFO | ATPRED | PLANDM | SR- | SR+ | L | **TOTAL** |
| SD | 0.29 | 0.20 | 0.04 | 0.05 | 0.06 | 0.23 | 0.06 | 0.05 | 0.01 | 1 |
| PI | 0.07 | 0.37 | 0.04 | 0.06 | 0.07 | 0.19 | 0.15 | 0.04 | 0.02 | 1 |
| GO | 0.11 | 0.25 | 0.14 | 0.03 | 0.10 | 0.18 | 0.12 | 0.06 | 0.01 | 1 |
| INFO | 0.07 | 0.15 | 0.02 | 0.30 | 0.05 | 0.12 | 0.22 | 0.05 | 0.02 | 1 |
| ATPRED | 0.11 | 0.23 | 0.05 | 0.04 | 0.21 | 0.14 | 0.14 | 0.04 | 0.03 | 1 |
| PLANDM | 0.17 | 0.19 | 0.01 | 0.05 | 0.04 | 0.43 | 0.08 | 0.03 | 0.00 | 1 |
| SR- | 0.03 | 0.21 | 0.03 | 0.08 | 0.06 | 0.08 | 0.43 | 0.05 | 0.03 | 1 |
| SR+ | 0.07 | 0.11 | 0.02 | 0.07 | 0.04 | 0.13 | 0.18 | 0.36 | 0.02 | 1 |
| L | 0.09 | 0.30 | 0.00 | 0.06 | 0.10 | 0.19 | 0.23 | 0.03 | 0.00 | 1 |
| TOTAL | 0.11 | 0.23 | 0.03 | 0.08 | 0.07 | 0.22 | 0.18 | 0.06 | 0.02 | 1 |
|  |  |  |  |  |  |  |  |  |  |  |
|  |  |  |  |  |  |  |  |  |  |  |
| EXPECTED FREQUENCIES | | |  |  |  |  |  |  |  |  |
|  | SD | PI | GO | INFO | ATPRED | PLANDM | SR- | SR+ | L | **TOTAL** |
| SD | 62.06 | 127.35 | 17.42 | 41.41 | 36.34 | 118.70 | 100.93 | 33.45 | 9.34 | 547.00 |
| PI | 120.94 | 248.18 | 33.94 | 80.70 | 70.81 | 231.32 | 196.70 | 65.19 | 18.21 | 1066.00 |
| GO | 18.04 | 37.02 | 5.06 | 12.04 | 10.56 | 34.50 | 29.34 | 9.72 | 2.72 | 159.00 |
| INFO | 39.37 | 80.79 | 11.05 | 26.27 | 23.05 | 75.30 | 64.03 | 21.22 | 5.93 | 347.00 |
| ATPRED | 33.81 | 69.38 | 9.49 | 22.56 | 19.80 | 64.67 | 54.99 | 18.22 | 5.09 | 298.00 |
| PLANDM | 122.19 | 250.74 | 34.30 | 81.54 | 71.54 | 233.71 | 198.73 | 65.86 | 18.40 | 1077.00 |
| SR- | 98.48 | 202.08 | 27.64 | 65.71 | 57.66 | 188.35 | 160.16 | 53.08 | 14.83 | 868.00 |
| SR+ | 34.15 | 70.08 | 9.58 | 22.79 | 19.99 | 65.32 | 55.54 | 18.41 | 5.14 | 301.00 |
| L | 8.96 | 18.39 | 2.52 | 5.98 | 5.25 | 17.14 | 14.58 | 4.83 | 1.35 | 79.00 |
| TOTAL | 538.00 | 1104.00 | 151.00 | 359.00 | 315.00 | 1029.00 | 875.00 | 290.00 | 81.00 | 4742.00 |
|  |  |  |  |  |  |  |  |  |  |  |
|  |  |  |  |  |  |  |  |  |  |  |
| z-scores |  |  |  |  |  |  |  |  |  |  |
|  | SD | PI | GO | INFO | ATPRED | PLANDM | SR- | SR+ | L |  |
| SD | 14.18 | -1.65 | 0.93 | -2.65 | -0.79 | 1.03 | -7.73 | -1.03 | -1.87 |  |
| PI | -5.59 | 11.76 | 1.40 | -2.20 | 0.45 | -2.47 | -3.20 | -3.66 | 1.29 |  |
| GO | -0.26 | 0.57 | 8.24 | -2.15 | 1.76 | -1.27 | -2.15 | -0.24 | -0.45 |  |
| INFO | -2.70 | -3.67 | -1.60 | 16.60 | -1.58 | -4.50 | 1.86 | -1.21 | 0.89 |  |
| ATPRED | 0.04 | 0.09 | 1.88 | -2.62 | 10.14 | -3.29 | -1.85 | -1.80 | 2.27 |  |
| PLANDM | 6.43 | -4.08 | -3.61 | -4.26 | -3.56 | 18.94 | -9.63 | -5.04 | -4.12 |  |
| SR- | -8.82 | -1.96 | -1.21 | 0.89 | -1.31 | -10.51 | 20.99 | -1.27 | 3.24 |  |
| SR+ | -2.66 | -5.23 | -0.88 | -0.18 | -1.91 | -3.66 | -0.24 | 22.27 | -0.07 |  |
| L | -0.70 | 1.51 | -1.63 | -0.42 | 1.25 | -0.59 | 1.00 | -1.34 | -1.18 |  |

Table 3

*Germany WINFIRE Contingency Table*

| OBSERVED FREQUENCIES | | |  |  |  |  |  |  |  |  |
| --- | --- | --- | --- | --- | --- | --- | --- | --- | --- | --- |
|  | SD | PI | GO | INFO | ATPRED | PLANDM | SR- | SR+ | L | TOTAL |
| SD | 70 | 68 | 34 | 37 | 36 | 113 | 21 | 21 | 1 | 401 |
| PI | 57 | 145 | 74 | 75 | 73 | 155 | 129 | 16 | 3 | 727 |
| GO | 25 | 41 | 64 | 24 | 34 | 126 | 27 | 10 | 2 | 353 |
| INFO | 56 | 71 | 20 | 127 | 39 | 95 | 46 | 16 | 4 | 474 |
| ATPRED | 45 | 97 | 20 | 45 | 68 | 91 | 36 | 16 | 1 | 419 |
| PLANDM | 98 | 177 | 69 | 131 | 108 | 450 | 74 | 43 | 1 | 1151 |
| SR- | 22 | 99 | 38 | 49 | 43 | 81 | 68 | 12 | 4 | 416 |
| SR+ | 19 | 33 | 10 | 21 | 12 | 30 | 10 | 8 | 0 | 143 |
| L | 2 | 7 | 1 | 4 | 0 | 1 | 2 | 0 | 0 | 17 |
| TOTAL | 394 | 738 | 330 | 513 | 413 | 1142 | 413 | 142 | 16 | 4101 |
|  |  |  |  |  |  |  |  |  |  |  |
| CONDITIONAL PROBABLITIES | | |  |  |  |  |  |  |  |  |
|  | SD | PI | GO | INFO | ATPRED | PLANDM | SR- | SR+ | L | TOTAL |
| SD | 0.17 | 0.17 | 0.08 | 0.09 | 0.09 | 0.28 | 0.05 | 0.05 | 0.00 | 1 |
| PI | 0.08 | 0.20 | 0.10 | 0.10 | 0.10 | 0.21 | 0.18 | 0.02 | 0.00 | 1 |
| GO | 0.07 | 0.12 | 0.18 | 0.07 | 0.10 | 0.36 | 0.08 | 0.03 | 0.01 | 1 |
| INFO | 0.12 | 0.15 | 0.04 | 0.27 | 0.08 | 0.20 | 0.10 | 0.03 | 0.01 | 1 |
| ATPRED | 0.11 | 0.23 | 0.05 | 0.11 | 0.16 | 0.22 | 0.09 | 0.04 | 0.00 | 1 |
| PLANDM | 0.09 | 0.15 | 0.06 | 0.11 | 0.09 | 0.39 | 0.06 | 0.04 | 0.00 | 1 |
| SR- | 0.05 | 0.24 | 0.09 | 0.12 | 0.10 | 0.19 | 0.16 | 0.03 | 0.01 | 1 |
| SR+ | 0.13 | 0.23 | 0.07 | 0.15 | 0.08 | 0.21 | 0.07 | 0.06 | 0.00 | 1 |
| L | 0.12 | 0.41 | 0.06 | 0.24 | 0.00 | 0.06 | 0.12 | 0.00 | 0.00 | 1 |
| TOTAL | 0.10 | 0.18 | 0.08 | 0.13 | 0.10 | 0.28 | 0.10 | 0.03 | 0.00 | 1 |
|  |  |  |  |  |  |  |  |  |  |  |
|  |  |  |  |  |  |  |  |  |  |  |
| EXPECTED FREQUENCIES | | |  |  |  |  |  |  |  |  |
|  | SD | PI | GO | INFO | ATPRED | PLANDM | SR- | SR+ | L | TOTAL |
| SD | 38.53 | 72.16 | 32.27 | 50.16 | 40.38 | 111.67 | 40.38 | 13.88 | 1.56 | 401 |
| PI | 69.85 | 130.83 | 58.50 | 90.94 | 73.21 | 202.45 | 73.21 | 25.17 | 2.84 | 727 |
| GO | 33.91 | 63.52 | 28.41 | 44.16 | 35.55 | 98.30 | 35.55 | 12.22 | 1.38 | 353 |
| INFO | 45.54 | 85.30 | 38.14 | 59.29 | 47.74 | 131.99 | 47.74 | 16.41 | 1.85 | 474 |
| ATPRED | 40.26 | 75.40 | 33.72 | 52.41 | 42.20 | 116.68 | 42.20 | 14.51 | 1.63 | 419 |
| PLANDM | 110.58 | 207.13 | 92.62 | 143.98 | 115.91 | 320.52 | 115.91 | 39.85 | 4.49 | 1151 |
| SR- | 39.97 | 74.86 | 33.47 | 52.04 | 41.89 | 115.84 | 41.89 | 14.40 | 1.62 | 416 |
| SR+ | 13.74 | 25.73 | 11.51 | 17.89 | 14.40 | 39.82 | 14.40 | 4.95 | 0.56 | 143 |
| L | 1.63 | 3.06 | 1.37 | 2.13 | 1.71 | 4.73 | 1.71 | 0.59 | 0.07 | 17 |
| TOTAL | 394 | 738 | 330 | 513 | 413 | 1142 | 413 | 142 | 16 | 4101 |
|  |  |  |  |  |  |  |  |  |  |  |
|  |  |  |  |  |  |  |  |  |  |  |
| z-scores |  |  |  |  |  |  |  |  |  |  |
|  | SD | PI | GO | INFO | ATPRED | PLANDM | SR- | SR+ | L |  |
| SD | 5.62 | -0.57 | 0.33 | -2.09 | -0.77 | 0.16 | -3.39 | 2.05 | -0.48 |  |
| PI | -1.78 | 1.51 | 2.33 | -1.97 | -0.03 | -4.33 | 7.58 | -2.05 | 0.11 |  |
| GO | -1.68 | -3.26 | 7.29 | -3.39 | -0.29 | 3.44 | -1.58 | -0.68 | 0.56 |  |
| INFO | 1.73 | -1.82 | -3.26 | 10.00 | -1.42 | -4.03 | -0.28 | -0.11 | 1.68 |  |
| ATPRED | 0.83 | 2.90 | -2.60 | -1.16 | 4.42 | -2.95 | -1.06 | 0.42 | -0.52 |  |
| PLANDM | -1.48 | -2.73 | -3.02 | -1.36 | -0.91 | 10.04 | -4.84 | 0.60 | -1.95 |  |
| SR- | -3.15 | 3.25 | 0.86 | -0.47 | 0.19 | -4.02 | 4.49 | -0.68 | 1.97 |  |
| SR+ | 1.52 | 1.61 | -0.47 | 0.80 | -0.68 | -1.87 | -1.24 | 1.42 | -0.76 |  |
| L | 0.30 | 2.49 | -0.33 | 1.38 | -1.38 | -2.02 | 0.23 | -0.78 | -0.26 |  |

Table 4

*Germany COLDSTORE Contingency Table*

| OBSERVED FREQUENCIES | | |  |  |  |  |  |  |  |  |
| --- | --- | --- | --- | --- | --- | --- | --- | --- | --- | --- |
|  | SD | PI | GO | INFO | ATPRED | PLANDM | SR- | SR+ | L | TOTAL |
| SD | 263 | 147 | 75 | 35 | 110 | 333 | 38 | 44 | 2 | 1047 |
| PI | 128 | 159 | 71 | 45 | 95 | 418 | 93 | 19 | 4 | 1032 |
| GO | 61 | 85 | 154 | 27 | 52 | 92 | 38 | 15 | 3 | 527 |
| INFO | 40 | 33 | 20 | 56 | 26 | 39 | 26 | 6 | 2 | 248 |
| ATPRED | 103 | 108 | 54 | 38 | 107 | 95 | 55 | 25 | 3 | 588 |
| PLANDM | 349 | 401 | 86 | 40 | 129 | 532 | 49 | 58 | 1 | 1645 |
| SR- | 51 | 74 | 32 | 29 | 48 | 54 | 44 | 25 | 3 | 360 |
| SR+ | 50 | 24 | 7 | 4 | 29 | 53 | 19 | 46 | 2 | 234 |
| L | 6 | 2 | 1 | 1 | 1 | 2 | 1 | 1 | 0 | 15 |
| TOTAL | 1051 | 1033 | 500 | 275 | 597 | 1618 | 363 | 239 | 20 | 5696 |
|  |  |  |  |  |  |  |  |  |  |  |
| CONDITIONAL PROBABLITIES | | |  |  |  |  |  |  |  |  |
|  | SD | PI | GO | INFO | ATPRED | PLANDM | SR- | SR+ | L | TOTAL |
| SD | 0.25 | 0.14 | 0.07 | 0.03 | 0.11 | 0.32 | 0.04 | 0.04 | 0.00 | 1 |
| PI | 0.12 | 0.15 | 0.07 | 0.04 | 0.09 | 0.41 | 0.09 | 0.02 | 0.00 | 1 |
| GO | 0.12 | 0.16 | 0.29 | 0.05 | 0.10 | 0.17 | 0.07 | 0.03 | 0.01 | 1 |
| INFO | 0.16 | 0.13 | 0.08 | 0.23 | 0.10 | 0.16 | 0.10 | 0.02 | 0.01 | 1 |
| ATPRED | 0.18 | 0.18 | 0.09 | 0.06 | 0.18 | 0.16 | 0.09 | 0.04 | 0.01 | 1 |
| PLANDM | 0.21 | 0.24 | 0.05 | 0.02 | 0.08 | 0.32 | 0.03 | 0.04 | 0.00 | 1 |
| SR- | 0.14 | 0.21 | 0.09 | 0.08 | 0.13 | 0.15 | 0.12 | 0.07 | 0.01 | 1 |
| SR+ | 0.21 | 0.10 | 0.03 | 0.02 | 0.12 | 0.23 | 0.08 | 0.20 | 0.01 | 1 |
| L | 0.40 | 0.13 | 0.07 | 0.07 | 0.07 | 0.13 | 0.07 | 0.07 | 0.00 | 1 |
| TOTAL | 0.18 | 0.18 | 0.09 | 0.05 | 0.10 | 0.28 | 0.06 | 0.04 | 0.00 | 1 |
|  |  |  |  |  |  |  |  |  |  |  |
|  |  |  |  |  |  |  |  |  |  |  |
| EXPECTED FREQUENCIES | | |  |  |  |  |  |  |  |  |
|  | SD | PI | GO | INFO | ATPRED | PLANDM | SR- | SR+ | L | TOTAL |
| SD | 193.19 | 189.88 | 91.91 | 50.55 | 109.74 | 297.41 | 66.72 | 43.93 | 3.68 | 1047 |
| PI | 190.42 | 187.16 | 90.59 | 49.82 | 108.16 | 293.15 | 65.77 | 43.30 | 3.62 | 1032 |
| GO | 97.24 | 95.57 | 46.26 | 25.44 | 55.24 | 149.70 | 33.59 | 22.11 | 1.85 | 527 |
| INFO | 45.76 | 44.98 | 21.77 | 11.97 | 25.99 | 70.45 | 15.80 | 10.41 | 0.87 | 248 |
| ATPRED | 108.50 | 106.64 | 51.62 | 28.39 | 61.63 | 167.03 | 37.47 | 24.67 | 2.06 | 588 |
| PLANDM | 303.53 | 298.33 | 144.40 | 79.42 | 172.41 | 467.28 | 104.83 | 69.02 | 5.78 | 1645 |
| SR- | 66.43 | 65.29 | 31.60 | 17.38 | 37.73 | 102.26 | 22.94 | 15.11 | 1.26 | 360 |
| SR+ | 43.18 | 42.44 | 20.54 | 11.30 | 24.53 | 66.47 | 14.91 | 9.82 | 0.82 | 234 |
| L | 2.77 | 2.72 | 1.32 | 0.72 | 1.57 | 4.26 | 0.96 | 0.63 | 0.05 | 15 |
| TOTAL | 1051 | 1033 | 500 | 275 | 597 | 1618 | 363 | 239 | 20 | 5696 |
|  |  |  |  |  |  |  |  |  |  |  |
|  |  |  |  |  |  |  |  |  |  |  |
| z-scores |  |  |  |  |  |  |  |  |  |  |
|  | SD | PI | GO | INFO | ATPRED | PLANDM | SR- | SR+ | L |  |
| SD | 6.16 | -3.81 | -2.04 | -2.48 | 0.03 | 2.70 | -4.02 | 0.01 | -0.97 |  |
| PI | -5.54 | -2.51 | -2.38 | -0.77 | -1.48 | 9.52 | 3.84 | -4.17 | 0.22 |  |
| GO | -4.27 | -1.25 | 17.41 | 0.33 | -0.48 | -5.85 | 0.83 | -1.62 | 0.89 |  |
| INFO | -0.96 | -2.02 | -0.41 | 13.34 | 0.00 | -4.53 | 2.71 | -1.43 | 1.24 |  |
| ATPRED | -0.62 | 0.15 | 0.37 | 1.95 | 6.45 | -6.96 | 3.12 | 0.07 | 0.69 |  |
| PLANDM | 3.43 | 7.79 | -6.03 | -5.38 | -4.14 | 4.20 | -6.68 | -1.61 | -2.36 |  |
| SR- | -2.17 | 1.23 | 0.08 | 2.95 | 1.83 | -5.83 | 4.69 | 2.69 | 1.60 |  |
| SR+ | 1.17 | -3.19 | -3.19 | -2.27 | 0.98 | -1.99 | 1.12 | 12.05 | 1.33 |  |
| L | 2.15 | -0.48 | -0.29 | 0.33 | -0.48 | -1.30 | 0.05 | 0.48 | -0.23 |  |

Table 5

*Philippines WINFIRE Contingency Table*

| OBSERVED FREQUENCIES | | |  |  |  |  |  |  |  |  |
| --- | --- | --- | --- | --- | --- | --- | --- | --- | --- | --- |
|  | SD | PI | GO | INFO | ATPRED | PLANDM | SR- | SR+ | L | TOTAL |
| SD | 8 | 14 | 6 | 16 | 5 | 8 | 8 | 2 | 6 | 73 |
| PI | 8 | 110 | 24 | 44 | 22 | 43 | 67 | 6 | 49 | 373 |
| GO | 4 | 11 | 13 | 12 | 9 | 23 | 10 | 2 | 3 | 87 |
| INFO | 12 | 40 | 14 | 76 | 18 | 44 | 61 | 11 | 21 | 297 |
| ATPRED | 4 | 24 | 4 | 19 | 15 | 17 | 22 | 1 | 9 | 115 |
| PLANDM | 16 | 44 | 10 | 44 | 19 | 118 | 45 | 6 | 9 | 311 |
| SR- | 2 | 79 | 9 | 55 | 25 | 33 | 8 | 84 | 30 | 325 |
| SR+ | 3 | 5 | 1 | 9 | 1 | 5 | 8 | 4 | 2 | 38 |
| L | 4 | 42 | 3 | 22 | 6 | 17 | 34 | 2 | 6 | 136 |
| TOTAL | 61 | 369 | 84 | 297 | 120 | 308 | 263 | 118 | 135 | 1755 |
|  |  |  |  |  |  |  |  |  |  |  |
| CONDITIONAL PROBABLITIES | | |  |  |  |  |  |  |  |  |
|  | SD | PI | GO | INFO | ATPRED | PLANDM | SR- | SR+ | L | TOTAL |
| SD | 0.11 | 0.19 | 0.08 | 0.22 | 0.07 | 0.11 | 0.11 | 0.03 | 0.08 | 1 |
| PI | 0.02 | 0.29 | 0.06 | 0.12 | 0.06 | 0.12 | 0.18 | 0.02 | 0.13 | 1 |
| GO | 0.05 | 0.13 | 0.15 | 0.14 | 0.10 | 0.26 | 0.11 | 0.02 | 0.03 | 1 |
| INFO | 0.04 | 0.13 | 0.05 | 0.26 | 0.06 | 0.15 | 0.21 | 0.04 | 0.07 | 1 |
| ATPRED | 0.03 | 0.21 | 0.03 | 0.17 | 0.13 | 0.15 | 0.19 | 0.01 | 0.08 | 1 |
| PLANDM | 0.05 | 0.14 | 0.03 | 0.14 | 0.06 | 0.38 | 0.14 | 0.02 | 0.03 | 1 |
| SR- | 0.01 | 0.24 | 0.03 | 0.17 | 0.08 | 0.10 | 0.02 | 0.26 | 0.09 | 1 |
| SR+ | 0.08 | 0.13 | 0.03 | 0.24 | 0.03 | 0.13 | 0.21 | 0.11 | 0.05 | 1 |
| L | 0.03 | 0.31 | 0.02 | 0.16 | 0.04 | 0.13 | 0.25 | 0.01 | 0.04 | 1 |
| TOTAL | 0.03 | 0.21 | 0.05 | 0.17 | 0.07 | 0.18 | 0.15 | 0.07 | 0.08 | 1 |
|  |  |  |  |  |  |  |  |  |  |  |
|  |  |  |  |  |  |  |  |  |  |  |
| EXPECTED FREQUENCIES | | |  |  |  |  |  |  |  |  |
|  | SD | PI | GO | INFO | ATPRED | PLANDM | SR- | SR+ | L | TOTAL |
| SD | 2.54 | 15.35 | 3.49 | 12.35 | 4.99 | 12.81 | 10.94 | 4.91 | 5.62 | 73 |
| PI | 12.96 | 78.43 | 17.85 | 63.12 | 25.50 | 65.46 | 55.90 | 25.08 | 28.69 | 373 |
| GO | 3.02 | 18.29 | 4.16 | 14.72 | 5.95 | 15.27 | 13.04 | 5.85 | 6.69 | 87 |
| INFO | 10.32 | 62.45 | 14.22 | 50.26 | 20.31 | 52.12 | 44.51 | 19.97 | 22.85 | 297 |
| ATPRED | 4.00 | 24.18 | 5.50 | 19.46 | 7.86 | 20.18 | 17.23 | 7.73 | 8.85 | 115 |
| PLANDM | 10.81 | 65.39 | 14.89 | 52.63 | 21.26 | 54.58 | 46.61 | 20.91 | 23.92 | 311 |
| SR- | 11.30 | 68.33 | 15.56 | 55.00 | 22.22 | 57.04 | 48.70 | 21.85 | 25.00 | 325 |
| SR+ | 1.32 | 7.99 | 1.82 | 6.43 | 2.60 | 6.67 | 5.69 | 2.55 | 2.92 | 38 |
| L | 4.73 | 28.59 | 6.51 | 23.02 | 9.30 | 23.87 | 20.38 | 9.14 | 10.46 | 136 |
| TOTAL | 61 | 369 | 84 | 297 | 120 | 308 | 263 | 118 | 135 | 1755 |
|  |  |  |  |  |  |  |  |  |  |  |
|  |  |  |  |  |  |  |  |  |  |  |
| z-scores |  |  |  |  |  |  |  |  |  |  |
|  | SD | PI | GO | INFO | ATPRED | PLANDM | SR- | SR+ | L |  |
| SD | 3.57 | -0.40 | 1.40 | 1.16 | 0.00 | -1.51 | -0.98 | -1.39 | 0.17 |  |
| PI | -1.58 | 4.52 | 1.68 | -2.98 | -0.81 | -3.45 | 1.82 | -4.45 | 4.45 |  |
| GO | 0.59 | -1.97 | 4.55 | -0.80 | 1.33 | 2.24 | -0.94 | -1.69 | -1.52 |  |
| INFO | 0.58 | -3.51 | -0.06 | 4.37 | -0.58 | -1.36 | 2.94 | -2.28 | -0.44 |  |
| ATPRED | 0.00 | -0.04 | -0.68 | -0.12 | 2.73 | -0.81 | 1.29 | -2.59 | 0.06 |  |
| PLANDM | 1.77 | -3.28 | -1.43 | -1.44 | -0.56 | 10.42 | -0.28 | -3.72 | -3.50 |  |
| SR- | -3.12 | 1.61 | -1.89 | 0.00 | 0.68 | -3.88 | -7.01 | 15.25 | 1.15 |  |
| SR+ | 1.50 | -1.20 | -0.63 | 1.12 | -1.04 | -0.72 | 1.06 | 0.95 | -0.57 |  |
| L | -0.35 | 2.94 | -1.47 | -0.24 | -1.17 | -1.61 | 3.41 | -2.55 | -1.49 |  |

Table 6

*Philippines COLDSTORE Contingency Table*

| OBSERVED FREQUENCIES | | |  |  |  |  |  |  |  |  |
| --- | --- | --- | --- | --- | --- | --- | --- | --- | --- | --- |
|  | SD | PI | GO | INFO | ATPRED | PLANDM | SR- | SR+ | L | TOTAL |
| SD | 38 | 34 | 15 | 19 | 17 | 18 | 25 | 7 | 12 | 185 |
| PI | 50 | 121 | 35 | 54 | 32 | 47 | 54 | 10 | 17 | 420 |
| GO | 11 | 33 | 12 | 14 | 7 | 15 | 14 | 5 | 3 | 114 |
| INFO | 24 | 59 | 6 | 87 | 30 | 17 | 49 | 6 | 17 | 295 |
| ATPRED | 14 | 39 | 10 | 22 | 49 | 12 | 27 | 3 | 20 | 196 |
| PLANDM | 23 | 35 | 8 | 21 | 22 | 61 | 17 | 9 | 4 | 200 |
| SR- | 15 | 64 | 11 | 62 | 35 | 18 | 65 | 4 | 24 | 298 |
| SR+ | 4 | 5 | 3 | 5 | 7 | 6 | 9 | 11 | 8 | 58 |
| L | 12 | 20 | 10 | 13 | 20 | 4 | 24 | 4 | 4 | 111 |
| TOTAL | 191 | 410 | 110 | 297 | 219 | 198 | 284 | 59 | 109 | 1877 |
|  |  |  |  |  |  |  |  |  |  |  |
| CONDITIONAL PROBABLITIES | | |  |  |  |  |  |  |  |  |
|  | SD | PI | GO | INFO | ATPRED | PLANDM | SR- | SR+ | L | TOTAL |
| SD | 0.21 | 0.18 | 0.08 | 0.10 | 0.09 | 0.10 | 0.14 | 0.04 | 0.06 | 1 |
| PI | 0.12 | 0.29 | 0.08 | 0.13 | 0.08 | 0.11 | 0.13 | 0.02 | 0.04 | 1 |
| GO | 0.10 | 0.29 | 0.11 | 0.12 | 0.06 | 0.13 | 0.12 | 0.04 | 0.03 | 1 |
| INFO | 0.08 | 0.20 | 0.02 | 0.29 | 0.10 | 0.06 | 0.17 | 0.02 | 0.06 | 1 |
| ATPRED | 0.07 | 0.20 | 0.05 | 0.11 | 0.25 | 0.06 | 0.14 | 0.02 | 0.10 | 1 |
| PLANDM | 0.12 | 0.18 | 0.04 | 0.11 | 0.11 | 0.31 | 0.09 | 0.05 | 0.02 | 1 |
| SR- | 0.05 | 0.21 | 0.04 | 0.21 | 0.12 | 0.06 | 0.22 | 0.01 | 0.08 | 1 |
| SR+ | 0.07 | 0.09 | 0.05 | 0.09 | 0.12 | 0.10 | 0.16 | 0.19 | 0.14 | 1 |
| L | 0.11 | 0.18 | 0.09 | 0.12 | 0.18 | 0.04 | 0.22 | 0.04 | 0.04 | 1 |
| TOTAL | 0.10 | 0.22 | 0.06 | 0.16 | 0.12 | 0.11 | 0.15 | 0.03 | 0.06 | 1 |
|  |  |  |  |  |  |  |  |  |  |  |
|  |  |  |  |  |  |  |  |  |  |  |
| EXPECTED FREQUENCIES | | |  |  |  |  |  |  |  |  |
|  | SD | PI | GO | INFO | ATPRED | PLANDM | SR- | SR+ | L | TOTAL |
| SD | 18.83 | 40.41 | 10.84 | 29.27 | 21.58 | 19.52 | 27.99 | 5.82 | 10.74 | 185 |
| PI | 42.74 | 91.74 | 24.61 | 66.46 | 49.00 | 44.30 | 63.55 | 13.20 | 24.39 | 420 |
| GO | 11.60 | 24.90 | 6.68 | 18.04 | 13.30 | 12.03 | 17.25 | 3.58 | 6.62 | 114 |
| INFO | 30.02 | 64.44 | 17.29 | 46.68 | 34.42 | 31.12 | 44.64 | 9.27 | 17.13 | 295 |
| ATPRED | 19.94 | 42.81 | 11.49 | 31.01 | 22.87 | 20.68 | 29.66 | 6.16 | 11.38 | 196 |
| PLANDM | 20.35 | 43.69 | 11.72 | 31.65 | 23.34 | 21.10 | 30.26 | 6.29 | 11.61 | 200 |
| SR- | 30.32 | 65.09 | 17.46 | 47.15 | 34.77 | 31.44 | 45.09 | 9.37 | 17.31 | 298 |
| SR+ | 5.90 | 12.67 | 3.40 | 9.18 | 6.77 | 6.12 | 8.78 | 1.82 | 3.37 | 58 |
| L | 11.30 | 24.25 | 6.51 | 17.56 | 12.95 | 11.71 | 16.79 | 3.49 | 6.45 | 111 |
| TOTAL | 191 | 410 | 110 | 297 | 219 | 198 | 284 | 59 | 109 | 1877 |
|  |  |  |  |  |  |  |  |  |  |  |
|  |  |  |  |  |  |  |  |  |  |  |
| z-scores |  |  |  |  |  |  |  |  |  |  |
|  | SD | PI | GO | INFO | ATPRED | PLANDM | SR- | SR+ | L |  |
| SD | 4.91 | -1.20 | 1.37 | -2.18 | -1.11 | -0.38 | -0.65 | 0.53 | 0.42 |  |
| PI | 1.33 | 3.92 | 2.45 | -1.89 | -2.93 | 0.49 | -1.48 | -1.02 | -1.75 |  |
| GO | -0.19 | 1.89 | 2.19 | -1.07 | -1.90 | 0.94 | -0.88 | 0.78 | -1.50 |  |
| INFO | -1.26 | -0.83 | -3.05 | 7.01 | -0.87 | -2.91 | 0.77 | -1.19 | -0.04 |  |
| ATPRED | -1.48 | -0.70 | -0.48 | -1.86 | 6.14 | -2.13 | -0.56 | -1.37 | 2.78 |  |
| PLANDM | 0.66 | -1.57 | -1.19 | -2.18 | -0.31 | 9.72 | -2.77 | 1.16 | -2.44 |  |
| SR- | -3.20 | -0.17 | -1.74 | 2.57 | 0.05 | -2.76 | 3.51 | -1.94 | 1.81 |  |
| SR+ | -0.84 | -2.48 | -0.23 | -1.53 | 0.10 | -0.05 | 0.08 | 7.02 | 2.64 |  |
| L | 0.23 | -1.01 | 1.46 | -1.22 | 2.15 | -2.46 | 1.97 | 0.29 | -1.02 |  |

Table 7

*United States WINFIRE Contingency Table*

| OBSERVED FREQUENCIES | | |  |  |  |  |  |  |  |  |
| --- | --- | --- | --- | --- | --- | --- | --- | --- | --- | --- |
|  | SD | PI | GO | INFO | ATPRED | PLANDM | SR- | SR+ | L | TOTAL |
| SD | 28 | 33 | 31 | 20 | 10 | 28 | 18 | 10 | 3 | 181 |
| PI | 21 | 112 | 40 | 20 | 38 | 75 | 109 | 20 | 4 | 439 |
| GO | 27 | 40 | 59 | 15 | 21 | 80 | 41 | 15 | 1 | 299 |
| INFO | 18 | 20 | 15 | 52 | 17 | 27 | 38 | 5 | 4 | 196 |
| ATPRED | 10 | 28 | 22 | 12 | 50 | 54 | 53 | 8 | 4 | 241 |
| PLANDM | 28 | 68 | 65 | 42 | 50 | 269 | 79 | 20 | 4 | 625 |
| SR- | 19 | 125 | 47 | 39 | 48 | 70 | 142 | 15 | 10 | 515 |
| SR+ | 17 | 18 | 8 | 8 | 10 | 14 | 17 | 18 | 0 | 110 |
| L | 2 | 3 | 1 | 0 | 4 | 6 | 15 | 0 | 3 | 34 |
| TOTAL | 170 | 447 | 288 | 208 | 248 | 623 | 512 | 111 | 33 | 2640 |
|  |  |  |  |  |  |  |  |  |  |  |
| CONDITIONAL PROBABLITIES | | |  |  |  |  |  |  |  |  |
|  | SD | PI | GO | INFO | ATPRED | PLANDM | SR- | SR+ | L | TOTAL |
| SD | 0.15 | 0.18 | 0.17 | 0.11 | 0.06 | 0.15 | 0.10 | 0.06 | 0.02 | 1 |
| PI | 0.05 | 0.26 | 0.09 | 0.05 | 0.09 | 0.17 | 0.25 | 0.05 | 0.01 | 1 |
| GO | 0.09 | 0.13 | 0.20 | 0.05 | 0.07 | 0.27 | 0.14 | 0.05 | 0.00 | 1 |
| INFO | 0.09 | 0.10 | 0.08 | 0.27 | 0.09 | 0.14 | 0.19 | 0.03 | 0.02 | 1 |
| ATPRED | 0.04 | 0.12 | 0.09 | 0.05 | 0.21 | 0.22 | 0.22 | 0.03 | 0.02 | 1 |
| PLANDM | 0.04 | 0.11 | 0.10 | 0.07 | 0.08 | 0.43 | 0.13 | 0.03 | 0.01 | 1 |
| SR- | 0.04 | 0.24 | 0.09 | 0.08 | 0.09 | 0.14 | 0.28 | 0.03 | 0.02 | 1 |
| SR+ | 0.15 | 0.16 | 0.07 | 0.07 | 0.09 | 0.13 | 0.15 | 0.16 | 0.00 | 1 |
| L | 0.06 | 0.09 | 0.03 | 0.00 | 0.12 | 0.18 | 0.44 | 0.00 | 0.09 | 1 |
| TOTAL | 0.06 | 0.17 | 0.11 | 0.08 | 0.09 | 0.24 | 0.19 | 0.04 | 0.01 | 1 |
|  |  |  |  |  |  |  |  |  |  |  |
|  |  |  |  |  |  |  |  |  |  |  |
| EXPECTED FREQUENCIES | | |  |  |  |  |  |  |  |  |
|  | SD | PI | GO | INFO | ATPRED | PLANDM | SR- | SR+ | L | TOTAL |
| SD | 11.66 | 30.65 | 19.75 | 14.26 | 17.00 | 42.71 | 35.10 | 7.61 | 2.26 | 181 |
| PI | 28.27 | 74.33 | 47.89 | 34.59 | 41.24 | 103.60 | 85.14 | 18.46 | 5.49 | 439 |
| GO | 19.25 | 50.63 | 32.62 | 23.56 | 28.09 | 70.56 | 57.99 | 12.57 | 3.74 | 299 |
| INFO | 12.62 | 33.19 | 21.38 | 15.44 | 18.41 | 46.25 | 38.01 | 8.24 | 2.45 | 196 |
| ATPRED | 15.52 | 40.81 | 26.29 | 18.99 | 22.64 | 56.87 | 46.74 | 10.13 | 3.01 | 241 |
| PLANDM | 40.25 | 105.82 | 68.18 | 49.24 | 58.71 | 147.49 | 121.21 | 26.28 | 7.81 | 625 |
| SR- | 33.16 | 87.20 | 56.18 | 40.58 | 48.38 | 121.53 | 99.88 | 21.65 | 6.44 | 515 |
| SR+ | 7.08 | 18.63 | 12.00 | 8.67 | 10.33 | 25.96 | 21.33 | 4.63 | 1.38 | 110 |
| L | 2.19 | 5.76 | 3.71 | 2.68 | 3.19 | 8.02 | 6.59 | 1.43 | 0.43 | 34 |
| TOTAL | 170 | 447 | 288 | 208 | 248 | 623 | 512 | 111 | 33 | 2640 |
|  |  |  |  |  |  |  |  |  |  |  |
|  |  |  |  |  |  |  |  |  |  |  |
| z-scores |  |  |  |  |  |  |  |  |  |  |
|  | SD | PI | GO | INFO | ATPRED | PLANDM | SR- | SR+ | L |  |
| SD | 5.13 | 0.48 | 2.78 | 1.64 | -1.85 | -2.67 | -3.33 | 0.92 | 0.51 |  |
| PI | -1.55 | 5.25 | -1.32 | -2.83 | -0.58 | -3.52 | 3.15 | 0.40 | -0.70 |  |
| GO | 1.94 | -1.74 | 5.20 | -1.95 | -1.49 | 1.37 | -2.64 | 0.74 | -1.51 |  |
| INFO | 1.63 | -2.61 | -1.52 | 10.07 | -0.36 | -3.37 | 0.00 | -1.20 | 1.04 |  |
| ATPRED | -1.52 | -2.31 | -0.93 | -1.75 | 6.34 | -0.46 | 1.07 | -0.72 | 0.60 |  |
| PLANDM | -2.28 | -4.62 | -0.47 | -1.23 | -1.37 | 13.10 | -4.89 | -1.43 | -1.57 |  |
| SR- | -2.83 | 4.95 | -1.45 | -0.29 | -0.06 | -5.96 | 5.23 | -1.63 | 1.57 |  |
| SR+ | 3.93 | -0.16 | -1.25 | -0.24 | -0.11 | -2.74 | -1.07 | 6.49 | -1.21 |  |
| L | -0.13 | -1.27 | -1.50 | -1.72 | 0.48 | -0.82 | 3.67 | -1.23 | 4.00 |  |

Table 8

*United States COLDSTORE Contingency Table*

| OBSERVED FREQUENCIES | | |  |  |  |  |  |  |  |  |
| --- | --- | --- | --- | --- | --- | --- | --- | --- | --- | --- |
|  | SD | PI | GO | INFO | ATPRED | PLANDM | SR- | SR+ | L | TOTAL |
| SD | 136 | 74 | 66 | 16 | 32 | 97 | 27 | 31 | 2 | 481 |
| PI | 55 | 126 | 60 | 35 | 37 | 147 | 89 | 21 | 2 | 572 |
| GO | 63 | 73 | 37 | 14 | 18 | 39 | 28 | 24 | 0 | 296 |
| INFO | 19 | 34 | 14 | 35 | 7 | 15 | 24 | 6 | 5 | 159 |
| ATPRED | 35 | 54 | 20 | 14 | 43 | 28 | 21 | 14 | 1 | 230 |
| PLANDM | 105 | 115 | 36 | 10 | 45 | 174 | 63 | 37 | 0 | 585 |
| SR- | 31 | 74 | 30 | 31 | 31 | 60 | 107 | 21 | 7 | 392 |
| SR+ | 25 | 24 | 13 | 8 | 19 | 35 | 29 | 34 | 2 | 189 |
| L | 3 | 1 | 0 | 7 | 2 | 1 | 3 | 1 | 0 | 18 |
| TOTAL | 472 | 575 | 276 | 170 | 234 | 596 | 391 | 189 | 19 | 2922 |
|  |  |  |  |  |  |  |  |  |  |  |
| CONDITIONAL PROBABLITIES | | |  |  |  |  |  |  |  |  |
|  | SD | PI | GO | INFO | ATPRED | PLANDM | SR- | SR+ | L | TOTAL |
| SD | 0.28 | 0.15 | 0.14 | 0.03 | 0.07 | 0.20 | 0.06 | 0.06 | 0.00 | 1 |
| PI | 0.10 | 0.22 | 0.10 | 0.06 | 0.06 | 0.26 | 0.16 | 0.04 | 0.00 | 1 |
| GO | 0.21 | 0.25 | 0.13 | 0.05 | 0.06 | 0.13 | 0.09 | 0.08 | 0.00 | 1 |
| INFO | 0.12 | 0.21 | 0.09 | 0.22 | 0.04 | 0.09 | 0.15 | 0.04 | 0.03 | 1 |
| ATPRED | 0.15 | 0.23 | 0.09 | 0.06 | 0.19 | 0.12 | 0.09 | 0.06 | 0.00 | 1 |
| PLANDM | 0.18 | 0.20 | 0.06 | 0.02 | 0.08 | 0.30 | 0.11 | 0.06 | 0.00 | 1 |
| SR- | 0.08 | 0.19 | 0.08 | 0.08 | 0.08 | 0.15 | 0.27 | 0.05 | 0.02 | 1 |
| SR+ | 0.13 | 0.13 | 0.07 | 0.04 | 0.10 | 0.19 | 0.15 | 0.18 | 0.01 | 1 |
| L | 0.17 | 0.06 | 0.00 | 0.39 | 0.11 | 0.06 | 0.17 | 0.06 | 0.00 | 1 |
| TOTAL | 0.16 | 0.20 | 0.09 | 0.06 | 0.08 | 0.20 | 0.13 | 0.06 | 0.01 | 1 |
|  |  |  |  |  |  |  |  |  |  |  |
|  |  |  |  |  |  |  |  |  |  |  |
| EXPECTED FREQUENCIES | | |  |  |  |  |  |  |  |  |
|  | SD | PI | GO | INFO | ATPRED | PLANDM | SR- | SR+ | L | TOTAL |
| SD | 77.70 | 94.65 | 45.43 | 27.98 | 38.52 | 98.11 | 64.36 | 31.11 | 3.13 | 481 |
| PI | 92.40 | 112.56 | 54.03 | 33.28 | 45.81 | 116.67 | 76.54 | 37.00 | 3.72 | 572 |
| GO | 47.81 | 58.25 | 27.96 | 17.22 | 23.70 | 60.38 | 39.61 | 19.15 | 1.92 | 296 |
| INFO | 25.68 | 31.29 | 15.02 | 9.25 | 12.73 | 32.43 | 21.28 | 10.28 | 1.03 | 159 |
| ATPRED | 37.15 | 45.26 | 21.72 | 13.38 | 18.42 | 46.91 | 30.78 | 14.88 | 1.50 | 230 |
| PLANDM | 94.50 | 115.12 | 55.26 | 34.03 | 46.85 | 119.32 | 78.28 | 37.84 | 3.80 | 585 |
| SR- | 63.32 | 77.14 | 37.03 | 22.81 | 31.39 | 79.96 | 52.45 | 25.36 | 2.55 | 392 |
| SR+ | 30.53 | 37.19 | 17.85 | 11.00 | 15.14 | 38.55 | 25.29 | 12.22 | 1.23 | 189 |
| L | 2.91 | 3.54 | 1.70 | 1.05 | 1.44 | 3.67 | 2.41 | 1.16 | 0.12 | 18 |
| TOTAL | 472 | 575 | 276 | 170 | 234 | 596 | 391 | 189 | 19 | 2922 |
|  |  |  |  |  |  |  |  |  |  |  |
|  |  |  |  |  |  |  |  |  |  |  |
| z-scores |  |  |  |  |  |  |  |  |  |  |
|  | SD | PI | GO | INFO | ATPRED | PLANDM | SR- | SR+ | L |  |
| SD | 7.90 | -2.59 | 3.51 | -2.55 | -1.20 | -0.14 | -5.47 | -0.02 | -0.70 |  |
| PI | -4.74 | 1.58 | 0.95 | 0.34 | -1.51 | 3.51 | 1.71 | -3.03 | -1.00 |  |
| GO | 2.53 | 2.28 | 1.90 | -0.84 | -1.29 | -3.25 | -2.09 | 1.21 | -1.47 |  |
| INFO | -1.48 | 0.56 | -0.28 | 8.97 | -1.72 | -3.53 | 0.65 | -1.42 | 4.02 |  |
| ATPRED | -0.40 | 1.51 | -0.41 | 0.18 | 6.22 | -3.22 | -1.97 | -0.24 | -0.42 |  |
| PLANDM | 1.32 | -0.01 | -3.04 | -4.75 | -0.31 | 6.27 | -2.07 | -0.16 | -2.19 |  |
| SR- | -4.77 | -0.43 | -1.30 | 1.90 | -0.08 | -2.69 | 8.70 | -0.96 | 3.01 |  |
| SR+ | -1.13 | -2.50 | -1.25 | -0.96 | 1.07 | -0.66 | 0.82 | 6.66 | 0.72 |  |
| L | 0.06 | -1.51 | -1.37 | 6.01 | 0.49 | -1.57 | 0.41 | -0.16 | -0.34 |  |
